# “I’m a paper and pencil person”: a qualitative descriptive study of potential barriers and facilitators to engagement with pre-operative total knee replacement education and prehabilitation digital interventions

**DOI:** 10.1186/s12891-025-08673-1

**Published:** 2025-07-04

**Authors:** Anna M. Anderson, Anthony C. Redmond, Judith Joseph, Gretl A. McHugh

**Affiliations:** 1 Leeds Institute of Rheumatic and Musculoskeletal Medicine, University of Leeds, Leeds, UK; 2 National Institute for Health and Care Research (NIHR) Leeds Biomedical Research Centre, Leeds, UK; 3 Leeds Institute of Health Sciences, University of Leeds, Leeds, UK; 4 NIHR HealthTech Research Centre in Accelerated Surgical Care, Leeds, UK; 5 Centre for Clinical and Community Applications of Health Psychology, University of Southampton, Southampton, UK; 6 School of Healthcare, University of Leeds, Leeds, UK

**Keywords:** Total knee replacement, Total knee arthroplasty, Pre-operative education, Prehabilitation, Digital interventions, Qualitative, Focus groups

## Abstract

**Background:**

Interest in using digital interventions to provide pre-operative total knee replacement (TKR) education and prehabilitation (health/wellbeing optimization) support is growing. Patient engagement with digital interventions tends to be poor; therefore, exploring the intended users’ perspectives during digital intervention development is vital. This study was part of a project focused on developing a pre-operative TKR education and prehabilitation digital intervention, the *‘Virtual Knee School’* (VKS), and aimed to explore patients’ perspectives of potential barriers/facilitators to engagement with the VKS to inform its development.

**Methods:**

This United Kingdom-based, qualitative descriptive study involved 14 purposively selected patients who were awaiting/had undergone TKR. Three online focus groups were conducted to explore patients’ perspectives of barriers and facilitators to engagement with the behaviors targeted by the VKS and digital features that could address the barriers/facilitators. The focus groups were audio-recorded, professionally transcribed, and analyzed inductively using reflexive thematic analysis. Three Patient and Public Involvement representatives were involved in aspects such as reviewing the recruitment materials and/or plain English summary of the study findings.

**Results:**

Two intersecting themes were developed. Theme 1, *‘Accounting for individual differences’*, suggests pre-operative TKR digital interventions should account for the impact of individual differences on engagement with digital technologies, pre-operative education and prehabilitation. Most participants felt a pre-operative TKR digital intervention would be valuable; however, a couple of older participants appeared reluctant to use digital technologies. Participants’ perspectives of specific digital features and pre-operative TKR education and prehabilitation also varied widely. Theme 2, *‘Tailoring to the pre-operative context’* highlights the importance of tailoring pre-operative TKR digital interventions to pre-operative contextual features, including physiological/psychological factors, social/occupational factors and limitations in pre-operative TKR care provision. Various digital features that could address these factors were identified.

**Conclusions:**

This study’s findings suggest pre-operative TKR digital interventions should account for individual differences and be tailored to the pre-operative TKR context. Given that some patients are reluctant to use digital technologies, also offering pre-operative TKR support in non-digital formats is essential. The findings have been used to inform a VKS prototype and could also be used to inform the development of other pre-operative TKR digital interventions.

**Supplementary Information:**

The online version contains supplementary material available at 10.1186/s12891-025-08673-1.

## Background

Total knee replacement (TKR) is a common elective orthopaedic procedure. In 2023, 116,845 primary knee replacement procedures were recorded on the National Joint Registry for England, Wales, Northern Ireland, the Isle of Man, and Guernsey [1]. TKR is usually undertaken to relieve the pain and other disabling symptoms of end-stage knee osteoarthritis (OA) [1, 2]. OA is a chronic disease in which the destructive and reparative processes of joint tissues are imbalanced, leading to structural alterations throughout the joint [2]. Estimates suggest over 4,538,000 people in the United Kingdom (UK) have knee OA [3]. Key risk factors for OA include increasing age, previous knee injury, female gender, and being overweight or obese [4]. Correspondingly, the average age of patients undergoing knee replacement is 69 years old, approximately 55% of patients undergoing TKR are female, and approximately 90% are overweight or obese [1, 5]. In the English National Health Service (NHS), patients often face a long wait for TKR surgery [6]. Service disruptions due to the COVID-19 pandemic have greatly compounded this issue [7]. Although most patients’ symptoms improve post-TKR, around 20% of patients have an unfavorable pain outcome [8], with many more experiencing other residual symptoms such as swelling and difficulty kneeling [9, 10].

The UK National Institute for Health and Care Excellence (NICE) recommends providing patients awaiting TKR with pre-operative education and advice on prehabilitation [11]. Prehabilitation involves optimizing patients’ health and wellbeing prior to surgery with the aim of improving their post-operative recovery [12, 13]. A multimodal approach to prehabilitation is generally advocated, which may include various strategies such as exercise programs, weight management, and alcohol reduction [12, 13].

Research suggests pre-operative TKR education and prehabilitation may have various benefits, including helping to set realistic patient expectations, facilitating patient engagement with their post-operative rehabilitation, and reducing length of hospital stay [14, 15]. Pre-operative TKR support has commonly been delivered via face-to-face group classes, often known as *‘knee schools’* (11, 16: p.118). However, there is growing interest in digital pre-operative TKR support, particularly since the start of the COVID-19 pandemic [17–19]. As well as enabling remote care delivery, digital interventions offer benefits such as providing more personalized care, increasing patient engagement, and saving time for patients and clinicians [18].

Despite their potential benefits, many currently available TKR digital interventions have important limitations. For example, the quality of TKR smartphone applications and YouTube videos is highly variable [20, 21] and the readability of online TKR education materials is often above the recommended level [22]. Furthermore, it is widely acknowledged that patient engagement with digital interventions tends to be poor, limiting their ability to support the desired behavior changes [23]. Rigorously developing digital interventions is vital to address these issues [23]. When planning a novel digital intervention, it is important to explore the intended users’ perspectives of the behaviors the intervention seeks to change [24]. Identifying barriers and facilitators to the target behaviors is particularly valuable to enable features addressing the barriers and facilitators to be incorporated into the intervention [24]. This can help to ensure that the intervention being developed is acceptable, engaging and supportive of the desired behaviour changes [24].

Previous studies have provided some insights into patients’ perspectives of behaviors that could be targeted by pre-operative TKR education and prehabilitation digital interventions. However, they present limitations in terms of their sample, scope of behaviors considered and/or transferability to the UK context. Robinson et al. [17] explored orthopaedic patients’ perspectives of digital technologies but only 28% of participants had experience of TKR. Similarly, in a qualitative study involving a mock pre-operative education and prehabilitation eHealth tool by Reid et al. [25, 26], only 18% of the sample were awaiting/had undergone TKR. Pellegrini et al. [27–29] and Webber et al. [30] explored factors that may affect engagement with healthy lifestyle behaviors before and after TKR but did not specifically address pre-operative education. Other studies/reviews exploring pre-operative TKR education have focused on patients’ education needs and experiences [31, 32], rather than barriers and facilitators to engagement with the education. The studies by Reid et al. [25, 26], Pellegrini et al. [27–29] and Webber et al. [30] were all based in the United States or Canada; therefore, their findings are not directly transferable to the UK context.

This study was the second phase of a doctoral research project focused on developing a UK-based, pre-operative TKR education and prehabilitation digital intervention, the *‘Virtual Knee School’* (VKS) [33]. The VKS was developed specifically for patients undergoing TKR because factors such as patient demographics, length of hospital stay, and recovery timescales vary between TKR and other orthopaedic procedures such as partial knee replacement.

This study sought to address gaps in existing literature by exploring patients’ perspectives of potential barriers and facilitators to engagement with the VKS and had two objectives.

1. To explore patients’ perspectives of barriers and facilitators to engagement with the behaviors targeted by the VKS.

2. To explore patients’ perspectives of digital features that could address barriers and facilitators to engagement with the VKS.

## Methods

### Design

**Fig. 1:**
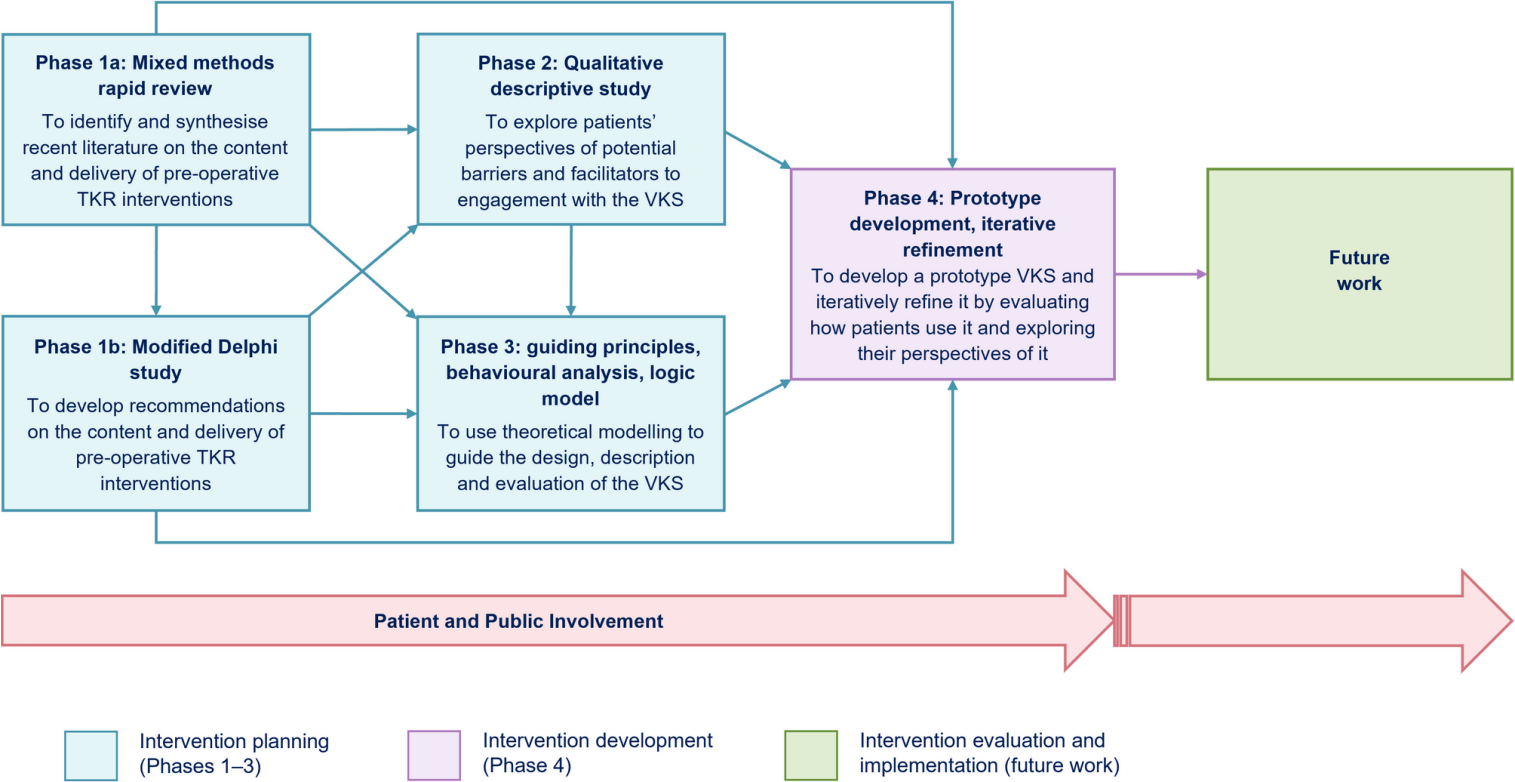
Flowchart of the overall Virtual Knee School development project. This figure has been reproduced from Fig. 1 in Anderson et al. [37] without any changes under the terms of the Creative Commons Attribution License (https://creativecommons.org/licenses/by/4.0/). An image description of Figure 1 is available in Additional File 1

The VKS development followed an evidence-, theory- and person-based approach [24, 34] and was informed by the 2006 version of the Medical Research Council (MRC) framework for developing and evaluating complex interventions [35]. The VKS project maps to the development stage of the MRC framework [35]. This highlights that primary research with the intended intervention users can be useful for developing a theoretical understanding of how a proposed intervention may achieve its intended changes [35]. The overall VKS project employed a complex mixed methods design, primarily underpinned by pragmatism [36]. This study was Phase 2 of the VKS project. Figure 1 summarizes the overall VKS development project and where this study fits. Further details of the VKS development project are reported in Anderson et al. [37].

In line with the person-based approach [24], exploratory qualitative research was conducted to gain in-depth insights into the contexts, needs and preferences of the intended VKS users. A qualitative descriptive design was chosen to help to ensure that the findings closely reflected the participants’ own perspectives [38]. This is particularly important when using qualitative findings for intervention development [39, 40].

### Registration, ethical approval and reporting

The VKS project was registered on the ISRCTN registry on 24th April 2020 (ISRCTN11759773). Ethical approval covering this study was obtained from the Yorkshire and The Humber - Bradford Leeds Research Ethics Committee (20/YH/0095). The Consolidated Criteria for Reporting Qualitative Research (COREQ) checklist [41] was used to guide this study’s reporting.

### Research team

The lead researcher (AMA) is a physiotherapist who undertook the study as part of a full-time Health Education England (HEE) / National Institute for Health and Care Research (NIHR) Clinical Doctoral Research Fellowship. She had experience of qualitative interviewing and facilitating group discussions. The other research team members’ expertise spans qualitative, quantitative and mixed methods research in diverse clinical areas, including orthopedics, digital health, musculoskeletal condition self-management, biomechanics, nursing and psychology.

### Project advisory group and patient and public involvement

The VKS project was overseen by a Project Advisory Group (PAG). Full PAG meetings were held approximately every six months throughout the 39-month project. The PAG included an independent chair, a collaborator from a local association of acute NHS trusts, three Patient and Public Involvement (PPI) representatives (one of whom was recruited after this study), the lead researcher (AMA) and three of her supervisors. The PPI representatives brought a range of relevant lived experiences to the study, such as lived experience of TKR and barriers to using digital technologies.

Key roles of the PAG in this specific study included discussing the project planning and setting and reviewing success criteria. In addition, the PPI representatives reviewed this study’s recruitment materials (e.g., Participant Information Sheet), Topic Guide, digital trigger materials (examples of website features shown to participants), and/or plain English summary of the study findings. This led to various changes, such as increasing the font size on the recruitment materials, including a pain tracker in the digital trigger materials, and amending the layout and wording of the plain English summary. Two PPI representatives were also involved in planning and co-presenting a public dissemination event, which included the findings of this study.

### Participants

The recruitment strategy and eligibility criteria were discussed and finalized with the PAG. A pragmatic approach was essential because the study was conducted in the early stages of the COVID-19 pandemic. Participants were recruited via Twitter, Facebook and emailing a brief overview of the study to patient participants in Phase 1 of the VKS project [42]. Individuals who heard about the study via word of mouth were also included. Recruitment via an NHS teaching hospital was planned but could not be undertaken due to COVID-19-related restrictions.

Individuals were eligible for inclusion if they were aged at least 18 years old; able to communicate in English; listed for primary TKR at a hospital in the UK and/or had undergone primary TKR at a hospital in the UK within the past two years; and able to use and access the Internet and email. Inclusion of patients unable to communicate in English was not possible because translation and interpreting funds were not available. The timeframe of two years for having undergone a TKR was chosen to ensure participants could remember their pre-operative experiences and is consistent with the approach used in Phases 1 and 4 of the VKS project [37, 42]. Individuals unable to give informed consent were excluded to ensure that all participants could engage in the data collection.

Recruiting a diverse range of participants is essential during intervention development studies to help ensure that the intervention is appropriate for as wide a spectrum of intended users as possible [24]. Correspondingly, maximum variation purposive sampling was employed to select participants who varied in key characteristics [43]. These comprised age, gender, self-reported confidence in using the Internet (unconfident, neither confident nor unconfident, confident, very confident [44]) and experience of TKR (listed for TKR versus undergone TKR). Participants were asked about these characteristics by the lead researcher at the screening stage.

There is no universally accepted approach for determining the sample size in qualitative studies [45, 46]. Aiming to achieve saturation is one of the most widely used approaches, with some authors considering it an important measure of quality in qualitative research [47]. Saturation may be broadly conceptualized as the point at which further data collection does not provide any new information [48]. However, additional qualitative data arguably always provide some new insights, and the concept of saturation is inconsistently defined, with various authors suggesting different types of saturation [45, 47]. Empirical tests of saturation typically involve counting codes and rely on a relatively fixed codebook [45, 49]. Such tests are incompatible with the fluid coding approach used in reflexive thematic analysis [45]. Correspondingly, Braun and Clarke [45] argue that data saturation is not a valid concept for all qualitative studies, particularly those that are highly interpretative. When data saturation is referred to, Braun and Clarke [45] recommend clarifying how it is conceptualized.

In line with this study’s qualitative descriptive design, it involved a relatively low level of interpretation (i.e., the focus was on ensuring that the findings closely reflected the participants’ own perspectives). Aiming to achieve saturation was therefore considered an appropriate approach for determining the sample size, but counting codes to assess saturation was not. Correspondingly, saturation was assessed subjectively and conceptualized as the point at which additional data collection was considered unlikely to lead to the identification of new themes, which may be considered to align with data or thematic saturation [47, 50]. This point was reached after completion of three focus groups. These included 14 participants in total, with four or five participants per group. None of the participants had received clinical care from the lead researcher, although one was a patient at the site where she was based. To help build rapport and minimize the risk of dropouts, the researcher discussed the study via telephone with all participants prior to their focus group.

### Data collection

Data were collected through focus groups to enable discussions between participants and reduce the facilitator’s influence, which can help to generate novel insights for guiding intervention development and enhance credibility [24, 51]. All participants were required to complete an eConsent Form and Questionnaire prior to participating in a focus group. The questionnaire was used to collect data about the participants’ characteristics, such as their ethnicity. Participants were primarily allocated to the focus groups based on when they were recruited and their availability/timing preferences, rather than their experience of TKR. This approach was chosen because ensuring the timing of focus groups is convenient for patients may encourage them to participate [52]. Furthermore, not all participants had been recruited at the time of the first and second focus groups as the recruitment overlapped with the data collection.

The lead researcher facilitated all the focus groups independently between 19th May 2020 and 3rd June 2020. No co-facilitators were involved due to the small size of the focus groups (four to five participants per group) and to ensure that participants could be offered maximum flexibility with the focus group scheduling. Due to COVID-19-related restrictions, the focus groups were held online using Blackboard Collaborate™, a secure videoconferencing tool. One participant in each of the focus groups joined via telephone. The remainder joined online. Given that some people do not feel comfortable joining group discussions online [53], participants were offered the option of participating in a telephone/online interview, but none chose that option. Two participants received family member assistance with using Blackboard Collaborate™. To the lead researcher’s knowledge, all the other participants were alone throughout their focus group.

Based on PPI representatives’ suggestions, the VKS was planned as a web-based intervention to maximize accessibility. Correspondingly, the focus groups explored the participants’ perspectives of barriers and facilitators to engagement with the following target behaviors:

- engagement with pre-operative TKR care in a web-based format (an essential precursor for the VKS to support other behaviour changes);
- engagement with pre-operative TKR education;
- engagement with a pre-operative TKR exercise program;
- engagement with healthy lifestyle changes.

The focus groups also explored digital features that could address the above barriers and facilitators. Digital features were not discussed for every individual barrier and facilitator, as that would have required the lead researcher to interrupt the natural flow of the discussions and resulted in the focus groups being excessively long.

The lead researcher facilitated each focus group using a Topic Guide (Additional File 2) based on the study objectives and previous relevant research [44, 54]. Two PPI representatives were invited to review the Topic Guide. The PPI representatives responded via email as organizing an in-person meeting to discuss the Topic Guide was not feasible within the study timeline. Neither of the PPI representatives suggested any changes to the Topic Guide, so no changes were made.

**Fig. 2:**
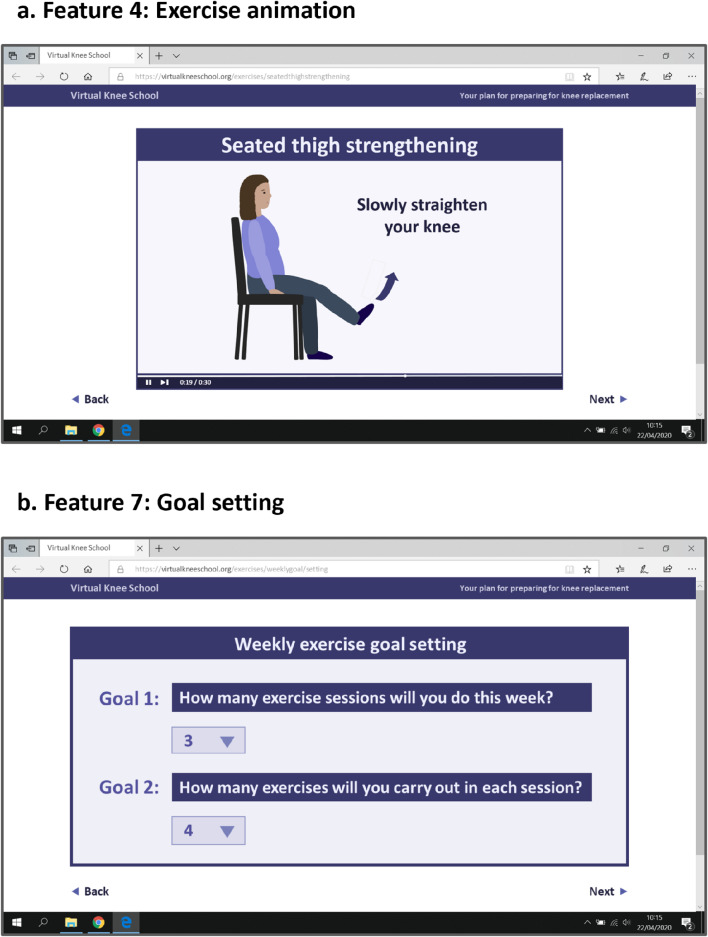
Trigger material examples. Two examples of the digital trigger materials discussed during the focus groups. (**a**) Image description. Feature 4: Exercise animation. Mock website screenshot of an exercise animation. The animation includes the title ‘Seated thigh strengthening’, an animated lady sat on a chair straightening one knee, an arrow in front of the lady’s foot pointing upwards, and the text ‘Slowly straighten your knee’. (**b**) Image description. Feature 7: Goal setting. Mock website screenshot of a weekly exercise goal-setting feature. Two goals are listed, with a dropdown menu below each goal. The Goal 1 text states ‘How many exercise sessions will you do this week?’ and ‘3’ is selected from the dropdown menu. The Goal 2 text states ‘How many exercises will you carry out in each session?’ and ‘4’ is selected from the dropdown menu

The first part of each focus group involved open-ended questions about participants’ experiences/perspectives of preparing for TKR and using TKR/health-related websites. To explore participants’ perspectives of website features they may not have been familiar with or mentioned spontaneously, the second part of each focus group involved discussing digital trigger materials [54] (Fig. 2). These consisted of 11 examples of website features developed based on the findings of Phase 1 of the VKS project [32, 42] and PPI representative feedback. The trigger materials were emailed to the participants as a Portable Document Format (PDF) document prior to their focus group and shared on screen during each focus group. Participants who joined the focus groups via telephone were directed to review the PDF document provided via email when the trigger materials were shared on screen.

The focus groups were all audio-recorded and transcribed intelligent verbatim. In line with good practice for qualitative research [41], the lead researcher recorded field notes during and/or shortly after each focus group. Key points recorded in the field notes related to the focus group practicalities (e.g., technical disruptions), group dynamics (e.g., whether all the participants contributed to similar degrees), and overall discussions (e.g., key points emphasized by participants).

### Data analysis

Data were analysed using reflexive thematic analysis [55, 56]. This is a flexible approach underpinned by qualitative research values [56], and hence was well suited for gaining an in-depth understanding of patients’ perspectives of potential barriers and facilitators to engagement with the VKS. Furthermore, reflexive thematic analysis can be used inductively [56], which is important for identifying unanticipated concerns about a proposed intervention [24, 57].

The lead researcher led the analysis by employing the six phases of reflexive thematic analysis flexibly [55, 56]. The six phases consisted of familiarization with the data collected; coding the data; generating initial themes and subthemes; developing and critically reviewing the themes and subthemes; refining, defining and naming the themes and subthemes; and writing an analytic narrative supported with data extracts [55, 56]. The coding was conducted using a fluid and largely semantic approach to help ensure the codes reflected participants’ perspectives. The critical review of the themes/subthemes led to redevelopment of all the themes/subthemes and collapsing of some themes/subthemes. A key change was collapsing two candidate themes, *‘Tailoring to pre-operative phase barriers’* and *‘Targeting key facilitators’*, into a single theme, *‘Tailoring to the pre-operative phase’*.

Field notes were used to assist the analysis where appropriate, with the summary of the overall focus group discussions being particularly useful for ensuring key points emphasized by participants were considered. QSR International NVivo software (Version 12 and Release 1) was employed to facilitate organization of the data. Additional File 3 provides an example of the theme/subtheme coding structure in the final NVivo file.

In addition to the narrative report, tables were created to summarize the barriers, facilitators and digital features from across both themes. This approach was based on a previous evidence-, theory- and person-based intervention planning study [58]. Separate tables were created for each behaviour targeted by the VKS to ensure the data were optimally structured for informing the subsequent project phases, which are described in Anderson et al. [37]. In contrast to the structured tables, the themes and subthemes were developed inductively as described above. This resulted in the tables not mapping exactly to the subthemes, and barriers and facilitators from the tables being interspersed across all six subthemes.

The lead researcher discussed the analysis with research team members at her monthly supervision meetings and via separate meetings/email correspondence as required. These discussions ensured differing perspectives were considered and helped resolve uncertainties that arose during the analysis process. For example, the lead researcher was initially unsure whether to create separate barriers and facilitators tables for engagement with pre-operative TKR care in a web-based format and engagement with pre-operative TKR education or combine them in a single table. Based on team discussions, it was decided to keep them separate because they are distinct concepts and combining them may have resulted in important details being lost.

Undertaking member checking was not considered a priority in this study given that the findings were used to inform a VKS prototype and feedback on the prototype was obtained through think-aloud interviews and PPI discussions, as reported in Anderson et al. [37].

### Trustworthiness and reflexivity

Multiple strategies were used to enhance trustworthiness, including addressing the four trustworthiness criteria proposed by Lincoln and Guba (Table 1) [59].

**Table 1:** Trustworthiness criteria and strategies used to address them

Criterion	Strategies^a^
Credibility	Focus groups were used to encourage participants to freely express their perspectives and enhance discussions through the group interaction of participants sharing similar or different views
	In line with standard practice, the researcher discussed the study via telephone with all the participants prior to their focus group to help establish rapport
	The findings were integrated with other data sources during the subsequent phases of the VKS project
	The findings informed the VKS prototype and feedback on the VKS prototype was obtained through think-aloud interviews in a subsequent phase of the VKS project [37]
Confirmability	Audio-recordings were transcribed by a professional transcription company then verified by the lead researcher
	Coding was inductive and focused on manifest content
	The lead researcher discussed the data analysis with the other research team members
	The lead researcher used a reflexive approach, including analysing the data using reflexive thematic analysis and keeping a reflexive journal
	Quotes are provided to support the themes and subthemes
Dependability	Detailed information is provided about the study procedures and no changes were made to the procedures during the study conduct
	An audit trail was maintained, including field notes, the reflexive journal and annotated NVivo files
Transferability	Detailed information is provided about the study design, context and participants
	Maximum variation purposive sampling was used to select a diverse range of relevant participants

*VKS*, Virtual Knee School

^a^ Strategies are allocated to the main criterion they correspond with but some strategies apply to more than one criterion

Table based on Lincoln and Guba [59], Given [43], Bradshaw et al. [82] and Milne and Oberle [51]

All the participants were aware that the researcher was a physiotherapist undertaking a PhD to develop a new pre-operative TKR care website. This did not appear to have a major impact on the data obtained as the participants generally appeared open about sharing their experiences of TKR care, both positive and negative. Similarly, participants were willing to express positive and negative perspectives about pre-operative TKR education, prehabilitation and digital interventions. The lead researcher was aware that she had developed various preconceptions about potential barriers and facilitators to engagement with the VKS through her clinical work, previous research, PPI activities and wider reading. To minimize the impact of these preconceptions on her interpretation of the data, she employed various strategies to enhance confirmability (Table 1). For example, she used a reflexive journal to record reflections on aspects such as her preconceptions, dual position as a researcher and health professional, key challenges encountered during the analysis process, and discussions with other research team members.

## Results

### Participants

Twenty-eight patients contacted the lead researcher to express an interest in participating, of whom 23 were screened, 17 were invited to participate and 15 consented. One consented participant did not join a focus group due to health problems. Reasons for exclusion at the screening stage were not meeting the eligibility criteria (*n* = 1), not meeting the purposive selection criteria (*n* = 4), and declining participation (*n* = 1). The flow chart in Additional File 4 provides further details of the flow of patients in the study.

The 14 participants who joined a focus group found out about the study via Twitter (*n* = 1), Facebook (*n* = 6), the study overview emailed to patient participants in Phase 1 of the VKS project (*n* = 5) or word of mouth (*n* = 2). Table 2 provides the focus group participants’ characteristics. The compositions of the focus groups were as follows:

- Focus group 1: three participants who were awaiting and had undergone TKR, and two participants who had undergone TKR.
- Focus group 2: one participant who was awaiting and had undergone TKR, three participants who had undergone one TKR, and one participant who had undergone TKR on both knees.
- Focus group 3: one participant who was awaiting TKR and three participants who had undergone TKR.

The focus groups lasted between 95 and 110 min. The illustrative quotes below are labelled with the participant’s pseudonym, age group, confidence in using the Internet and experience of TKR surgery.

**Table 2:** Focus group participant characteristics

	Number of participants (%) (*n* = 14)
**Age (years)**	
40–49	2 (14)
50–59	5 (36)
60–69	3 (21)
70–79	4 (29)
**Gender**	
Female	8 (57)
Male	6 (43)
**Confidence in using the Internet**	
Very confident	8 (57)
Confident	5 (36)
Unconfident	1 (7)
**Experience of TKR**	
Pre	1 (7)
Post	8 (57)
Pre, post	4 (29)
Post x 2	1 (7)
**Indication for TKR** ^**a**^	
Osteoarthritis	19 (100)
**Location of TKR** ^**a**^	
NHS hospital	12 (63)
Private hospital	7 (37)
**Months since previous TKR** ^**b**^	
< 3	3 (21)
3 < 6	3 (21)
6 < 12	2 (14)
12 < 24	4 (29)
≥ 24	2 (14)
**Body mass index (kg/m²)**	
18 < 25	1 (7)
25 < 30	7 (50)
30 < 40	5 (36)
≥ 40	1 (7)
**Ethnicity**	
White British	14 (100)
**Disability or health condition that could affect ability to use a website or carry out gentle exercises**	
Relevant disability/health condition	0 (0)
**Living location**	
Scotland	2 (14)
Northern Ireland	1 (7)
Wales	1 (7)
North East	1 (7)
North West	5 (36)
Yorkshire and the Humber	2 (14)
South East	2 (14)
**Highest educational qualification**	
None	1 (7)
GCSE/O-Level (or equivalent)	2 (14)
A-Level (or equivalent)	3 (21)
Vocational qualification	4 (29)
Undergraduate degree	2 (14)
Postgraduate degree	2 (14)
**Current employment status** ^**c**^	
Employed full-time	5 (36)
Self-employed	1 (7)
Retired	8 (57)
Medically disabled	1 (7)

*NHS*, National Health Service; *Post*, previously undergone TKR; *Post x 2*, previously undergone TKR on both knees; *Pre*, listed for TKR; *TKR*, total knee replacement

^a^ Participants who had undergone two TKRs/were both awaiting and had undergone TKR were counted twice (19 TKRs in total)

^b^ Only includes participants who had previously undergone TKR (*n* = 13 participants; 14 TKRs in total)

^c^ Participants could select more than one option

### Thematic analysis overview

**Fig. 3:**
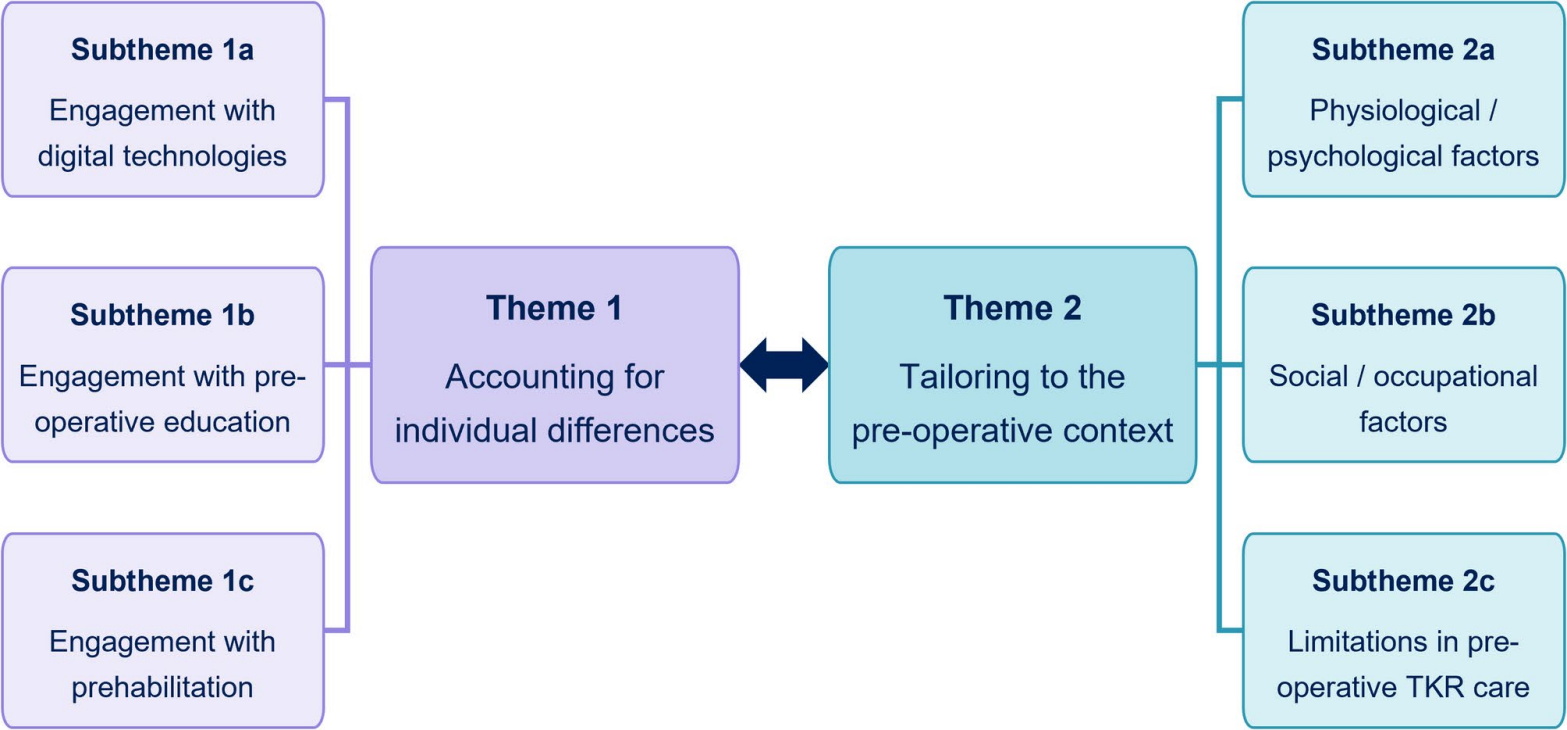
Themes and subthemes. *TKR*, total knee replacement. Summary of the two main themes and six subthemes. Image description. Two boxes labelled as ‘Theme 1: Accounting for individual differences’ and ‘Theme 2: Tailoring to the pre-operative context’ are in the middle of the figure joined by a double headed arrow. The Theme 1 box is joined by plain lines to three additional boxes labelled as ‘Subtheme 1a: Engagement with digital technologies’, ‘Subtheme 1b: Engagement with pre-operative education’ and ‘Subtheme 1c: Engagement with prehabilitation’. The Theme 2 box is joined by plain lines to three additional boxes labelled as ‘Subtheme 2a: Physiological/psychological factors’, ‘Subtheme 2b: Social/occupational factors’ and ‘Subtheme 2c: Limitations in pre-operative TKR care’

Two intersecting themes, each with three subthemes, were developed (Fig. 3). Each theme encapsulates a broad principle related to multiple potential barriers and facilitators to engagement with the VKS.

### Theme 1: accounting for individual differences

Multiple participants from across all three focus groups emphasized that *“everybody’s different”*. Differences in participants’ individual circumstances and preferences meant their perspectives of potential barriers and facilitators to engagement with the VKS varied. This theme includes three subthemes that demonstrate the impact of individual differences on barriers and facilitators to engagement with digital technologies, pre-operative education and prehabilitation. The subthemes highlight how digital features could address the individual differences where applicable. Overall, this theme suggests that accounting for individual differences would help to optimize patient engagement with pre-operative TKR education and prehabilitation digital interventions.

#### Subtheme 1a: engagement with digital technologies

Participants’ individual circumstances and preferences strongly influenced their perspectives of barriers and facilitators to engagement with digital technologies. Most participants liked the idea of a pre-operative TKR website, with participants in one focus group suggesting that a mobile application version would be valuable. However, one participant aged in her 70s commented that she is *“not up with all those sorts of apps and things”* and highlighted that limited experience of using digital tools is a currently an important barrier due to the older demographic of patients undergoing TKR. Correspondingly, another couple of participants aged in their 70s expressed a general reluctance to use digital technologies:

*I don’t own a watch*,* I don’t own a mobile phone. I’m 75*,* and I’m very happy like that.* (Lloyd, 70–79, unconfident, post-TKR)

*I’m a paper and pencil person*,* rather than technology*,* that’s it!* (Dorothy, 70–79, confident, post-TKR)

Dorothy and other participants appeared more willing to use a website if it was from a UK-based credible source, with features that are quick and simple to use. The opportunity to access specific digital features also appeared to be a facilitator to engagement with digital technologies, whilst concerns about specific digital features were a barrier. In many cases, participants’ perspectives of the same digital feature were directly opposing. For example, most participants in two focus groups preferred real-life videos to animations for exercise demonstrations:

*I think the one that I mentioned*,* the one the physio sent me*,* which was real people*,* and I think that helps better than doing an animation or*,* you know*,* just a stick figure on a piece of paper.* (Sophia, 40–49, very confident, post-TKR)

In contrast, participants in another focus group preferred animations, with one reporting a negative experience of a real-life video in a digital video disc (DVD) provided by her care team:

*Only the real person [in the DVD] was obviously a fit*,* healthy person who was just going through the exercises*,* and I found it very patronizing*,* that someone who was obviously fully capable of doing these exercises was showing me how to do them.* (Olivia, 50–59, very confident, post-TKR)

To account for individuals’ differing preferences, participants from all three focus groups felt that a pre-operative TKR care website should be flexible. Suggestions included making website features optional, making content such as an exercise diary available as a printable document, and allowing users to specify times for receiving email reminders:

*So I think something that actually pings up and says*,* oi*,* go and do*,* you know*,* ten stand and sits*,* now*,* to me*,* is probably more useful than having a notification coming through say in an email*,* that goes*,* open an email and it says*,* you should be doing this now.* (Jacob, 50–59, very confident, post-TKR)

#### Subtheme 1b: engagement with pre-operative education

Participants’ differing circumstances and preferences affected their willingness to engage with pre-operative education. A desire for detailed information about preparing for TKR and what to expect appeared to be an important facilitator to engagement with pre-operative education for most participants. Participants felt such information would help relieve their anxieties about their upcoming procedure and enable them to make practical preparations:

*I think a bit more advice on how to prepare the home for when you came back from hospital*,* certainly for me*,* would have been useful if I’d had that before the op.* (Irene, 50–59, confident, post-TKR)

However, one participant reported that he did not want detailed information pre-operatively, preferring to *“just get on with it”*. Differences were also evident in participants’ desire to find out about the TKR surgical procedure. A few participants chose to watch a video of TKR surgery, whereas most did not want to receive information about what would happen during their surgery:

*I did not want to know at all what they were going to do to me. And anybody who started to tell me*,* I just switched off*,* I did not want to know or to see anything*,* or to read about it or see pictures.* (Dorothy, 70–79, confident, post-TKR)

The risk of seeing graphic details, *“like a scene from a horror film”*, was a particular concern and presented a barrier to engagement with online information. Watching an animation was highlighted as a useful, less graphic alternative:

*It wasn’t obviously as graphic*,* it was sort of like an animation. So it just showed the knee open*,* but you couldn’t see anything graphic. So that was really good. But I think I would struggle with the graphics a bit.* (Sophia, 40–49, very confident, post-TKR)

Ensuring that information delivery approaches account for individuals’ differing needs was also considered important. Participants from one focus group highlighted low literacy levels and language barriers as potential barriers to engagement with pre-operative TKR education. These participants felt a website should include pictures, videos, and *“super simple”* language. Conversely, one participant was frustrated by receiving a large volume of simple information:

*The other thing we were given was*,* I think*,* about a 24-page booklet on what the operation would involve and how to recover. And again*,* it was an awful lot of simple information. So I found that a bit frustrating.* (Luke, 60–69, very confident, pre- and post-TKR)

#### Subtheme 1c: engagement with prehabilitation

As for engagement with digital technologies and pre-operative education, participants’ individual circumstances and preferences affected their perspectives of barriers/facilitators to engagement with prehabilitation. Differences in participants’ preferred exercise types and delivery modes appeared to be particularly important. For example, one participant reported finding exercise classes motivating, whereas another indicated that she does not like the constraints of exercise classes:

*Because I’m not good at following patterns*,* I’m not a*,* you know*,* I don’t like to have to*,* I don’t like classes*,* you know*,* I don’t like the routine*,* but I do like the exercises. So doing them as I felt like it*,* would work for me better. Just different people have different ways of doing it.* (Rosie, 60–69, very confident, pre- and post-TKR)

Lifestyle choices (e.g. being vegan), personal characteristics (e.g. a determined personality), and other health issues (e.g. heart problems) and were all perceived to affect engagement with prehabilitation:

*So I was all prepped up ready and then they said hang on*,* your heartrate’s too low. That’s because of all the physical exercise I do. So I had to slow down my exercise so they were going to go back four months later and have my operation done then.* (Cameron, 60–69, very confident, post-TKR)

Participants in one group suggested signposting patients to healthy eating advice on existing credible websites, as they felt that would account for individuals’ differing needs whilst avoiding unnecessary duplication of online information:

*And some people would say*,* you know*,* I can’t do this*,* or I’ve got that*,* and if you just referred them to the NHS page*,* it covers pretty much*,* basically everything*,* and all different*,* you know*,* eventualities of being vegan*,* or you know*,* diabetic*,* or anything like that.* (Sophia, 40–49, very confident, post-TKR)

Additional barriers to engagement with prehablitation related to participants’ differing environmental circumstances. Lack of access to specific equipment/facilities was identified as a barrier to engagement with exercise:

*I’d been going to the gym four or five times a week*,* and swimming*,* but then obviously lockdown happened*,* and I’ve not done as much.* (Molly, 40–49, very confident, pre-TKR)

Correspondingly, participants in one focus group emphasized the value of exercises that require household items only. Another environmental circumstance that presented a barrier to engagement with prehabilitation was going on holiday pre-operatively:

*The one thing that I did do differently*,* and it was particularly difficult for me*,* because we went on holiday*,* got back about ten days before the operation*,* and it was cutting down on alcohol. Now*,* I’m not an alcoholic*,* but I do like my wine at weekends*,* and on holiday.* (Irene, 50–59, confident, post-TKR)

### Theme 2: tailoring to the pre-operative context

Many of the reported barriers and facilitators to engagement with the behaviors targeted by the VKS appeared to relate closely to the pre-operative context. Key pre-operative contextual features included physiological/psychological factors, social/occupational factors, and limitations in pre-operative TKR care provision. This theme includes three subthemes that highlight barriers/facilitators and associated digital features related to these pre-operative contextual features. Overall, this theme suggests that tailoring pre-operative TKR digital interventions to these contextual features would help to optimize patients’ engagement with them.

Whilst this theme encompasses barriers and facilitators that appear to be common in the pre-operative TKR context, the barriers and facilitators do not apply to all individuals and may be experienced to differing degrees. Correspondingly, this theme intersects with Theme 1, *‘Accounting for individual differences’*.

#### Subtheme 2a: physiological/psychological factors

Certain physiological/psychological factors appeared to be particularly relevant during the pre-operative phase of the TKR pathway. Pre-operative knee signs/symptoms, such as pain, loss of movement and swelling, were a key barrier to engagement with prehabilitation:

*The only one I had was pain and it got so bad that I had to stop [exercising].* (Luke, 60–69, very confident, pre- and post-TKR)

Despite having to stop certain exercises completely, participants identified various strategies that enabled them to continue being physically active pre-operatively. These included using walking aids, activity pacing and performing non-weight-bearing activities. Knee signs/symptoms influenced the digital features that participants felt should be included in a pre-operative TKR care website. For example, some participants felt that pain and/or activity trackers would be more useful post-operatively than pre-operatively:

*I think I agree with what they’ve said*,* [tracking pain] afterwards would be more*,* probably*,* beneficial. Because at the moment*,* it’s mainly like in the high [pain] level all the time*,* so you don’t see the lower level that often*,* to be honest.* (Molly, 40–49, very confident, pre-TKR)

Prior to receiving specific advice, one participant was concerned that exercising pre-operatively could cause further knee damage. Overall however, participants’ beliefs appeared to be key psychological factors that facilitated their engagement with prehabilitation. Participants from all three focus groups seemed to be motivated to engage with pre-operative exercise and/or healthy lifestyle changes by the belief that doing so would improve their post-operative recovery:

*I know this might sound really counterintuitive but I said to myself*,* if I don’t do exercise or carry on doing some exercise*,* when I get to post-op my knee…I might not have any sufficient muscles or anything else around my knee to help me. So I said to myself I’ve got to just keep it going […].* (Cameron, 60–69, very confident, post-TKR)

Participants also perceived other benefits of prehabilitation, such as preventing their symptoms deteriorating, changing their appearance, and loosening other joints:

*I was just finding that when I was doing the exercises*,* I was finding it very*,* very difficult*,* but the more I did*,* the more it loosened up my other joints and I found it very*,* very helpful.* (Ivy, 50–59, confident, post-TKR on both knees)

Correspondingly, participants highlighted that they would be more motivated to engage with prehabilitation if they understood the reasons for doing so, and suggested that advice on prehabilitation should explain the benefits of specific exercises and healthy lifestyle changes, including in relation to TKR outcomes where appropriate:

*I think perhaps something in place to link between weight management and*,* you know*,* the joint replacement surgery*,* how that has an impact on how long it will last*,* how well it will function.* (Jacob, 50–59, very confident, post-TKR)

#### Subtheme 2b: social/occupational factors

Various pre-operative social/occupational factors presented barriers and/or facilitators to engagement with the behaviors targeted by the VKS. Participants highlighted how being busy with work and other distractions could prevent them from exercising or engaging with certain website features pre-operatively, whereas they were highly focused on their recovery in the early post-operative phase. Correspondingly, exercise reminders were considered more helpful pre- than post-operatively:

*Yes*,* I think so because once you’ve had the operation*,* you know you’ve got to exercise and initially all you’re doing all day is exercising and taking tablets. So you know that’s an easy routine to get into. I had a timetable as well. But beforehand when life was much more normal and very full*,* yes*,* I think reminders would be useful.* (Dorothy, 70–79, confident, post-TKR)

Social factors also presented facilitators to engagement with prehabilitation. Accountability to and feedback from health professionals appeared to be particularly important, with one participant reporting that his main motivation for losing weight pre-operatively was *“a massive rollicking from the consultant”*. Correspondingly, some participants felt recording their goals/exercises on a website for health professionals to view would be valuable:

*When I looked at your proforma thing*,* you know*,* your suggestions of things you might put on your website*,* I thought giving you the exercises and then sort of saying*,* did you manage to do it this week*,* and that sort of thing*,* that would be quite helpful*,* that sort of feedback. Because I found seeing the physio was a good motivation*,* I need somebody to sort of say*,* have you done it*,* you know.* (Beatrice, 70–79, confident, pre- and post-TKR)

Peer influences also appeared to be important, with participants from all focus groups reporting it was helpful to chat with previous patients informally, for example via a social media discussion group or when meeting in person:

*And so you can have a chat to him and find out what problems he had and how good it was and how much pain he was in. So it does give you an insight into what to expect when you are going to have the operation. So the more people you can see or meet*,* I think it’s the better.* (Harry, 70–79, confident, post-TKR)

Most participants’ desire to find out about other patients’ experiences of TKR appeared to be a facilitator to engagement with pre-operative TKR education. However, some participants expressed concerns about making comparisons with other patients on social media or finding out *“horror stories”* of TKR surgery:

*I had the mother*,* who had gone through the experience*,* and there was a friend who goes to a support group that I go to*,* who had the horror story from hell of her experience. And if I heard it once*,* I heard it three dozen times*,* from her*,* of what had happened to her. So*,* no*,* I didn’t use Google*,* because I didn’t want to find out more horror stories.* (Olivia, 50–59, very confident, post-TKR)

#### Subtheme 2c: limitations in pre-operative TKR care

Participants’ experiences of pre-operative TKR care were highly varied. Whilst some had positive experiences, many participants highlighted limitations in the pre-operative care they received. These limitations presented barriers to participants’ pre-operative preparations. Being unable to access adequate pre-operative support through other sources also appeared to be a potential facilitator to engagement with a pre-operative TKR website. A key pre-operative care limitation mentioned by participants from all three focus groups was lack of guidance on pre-operative exercise:

*But there was nothing that I got in terms of here’s exercises that you should do*,* or anything like that at all*,* it was very*,* very vague. That’s just what seems to be the problem*,* really*,* there’s no common approach to it*,* everybody just seemed to sort of do their own thing.* (James, 50–59, very confident, pre- and post-TKR)

Similarly, participants highlighted various deficiencies in the pre-operative TKR education they received, such as inadequate information on how to practically prepare for surgery and how much pain to expect post-operatively. Participants felt it would be helpful for a pre-operative TKR website to address these limitations, for example by providing a home preparations checklist:

*It could be good to have a checklist of things you need to get prepared at home before you go in. Because you don’t always get the information from your physio or your knee school at the hospital.* (Molly, 40–49, very confident, pre-TKR)

Participants also highlighted problems related to the timing of pre-operative TKR care delivery. One participant received a comprehensive information booklet, but felt it was not provided long enough before her surgery:

*So when I got my package with*,* it was a very well prepared booklet thing that I was given*,* with a color coded card for each aspect of the operation*,* and exercise*,* and everything*,* all in a nice folder I was given. But it wasn’t really long enough beforehand*,* in a way.* (Beatrice, 70–79, confident, pre- and post-TKR)

Another did not receive any pre-operative education or prehabilitation support because she underwent TKR at short notice due to a cancellation. Both these participants felt that a resource like a website could help address these issues by providing rapid access to information:

*But I knew nothing about what was going to happen or anything at all*,* no. It would be super if*,* as a result of this whole PhD that you’re doing Anna*,* if there could be some means of either a DVD or booklet or something*,* or website*,* of giving information to people in that position so that in a short space of time*,* at least you’ve got the knowledge.* (Dorothy, 70–79, confident, post-TKR)

### Barriers and facilitators tables

Tables 3, 4, 5 and 6 present summaries of the tables used to record all the identified barriers and facilitators to engagement with the behaviors targeted by the VKS and design features that could address the barriers and facilitators. Only design features discussed in the focus groups are included in the tables. Additional ideas for the design features were subsequently generated through research team discussions and are reported in Anderson et al. [37].

**Table 3:** Overview of barriers and facilitators to engagement with pre-operative total knee replacement care in a web-based format

Barriers to the target behaviour	Facilitators to the target behaviour	VKS design features that could address the barrier and/or facilitator
Limited experience of using digital tools	N/A	None discussed
Reluctance to use digital technologies Concerns about the reliability of websites	N/A	Indication the VKS is credible and UK-based Self-monitoring tool recording sheets that users can download and print out
Concerns about finding out about *“horror stories”* of TKR surgery	N/A	None discussed
Concerns about seeing graphic details of TKR surgery	N/A	Animation of the TKR surgical procedure, which does not show any graphic details
Concerns about the detail/duration of website interactions	N/A	Short videos/animations Digital tools with quick simple recording
Being busy with other commitments/distractions	N/A	None discussed
Concerns about specific digital features	Opportunity to access specific digital features	Feature optionality Videos of the TKR surgical procedure; videos of people performing practical tasks; videos of people performing exercises; animations of people performing exercises; online discussion group; trackers/diaries for exercise, physical activity, eating habits or pain; goal setting, review and feedback feature; quizzes; checklists; and exercise email reminders
N/A	Lack of timely access to adequate pre-operative TKR education and prehabilitation from other sources	Information, exercise guidance and healthy lifestyle guidance Information that can be accessed rapidly Indication the VKS is credible and UK-based
N/A	Signposting from health professionals	This facilitator cannot be directly addressed through the VKS itself
N/A	Publicity	This facilitator cannot be directly addressed through the VKS itself
N/A	Family member support	None discussed

*N/A*, not applicable; *TKR*, total knee replacement; *UK*, United Kingdom; *VKS*, Virtual Knee School

**Table 4:** Overview of barriers and facilitators to engagement with pre-operative total knee replacement education

Barriers to the target behaviour	Facilitators to the target behaviour	VKS design features that could address the barrier and/or facilitator
Short length of time between being listed for TKR surgery and undergoing TKR surgery	N/A	Information that can be accessed rapidly
Low literacy and language barriers	N/A	Simple language, pictures and videos
Reluctance to receive detailed pre-operative information	Desire for detailed information about preparing for TKR surgery and what to expect	Videos of real people performing practical tasks, including using walking aids, getting in/out of a car, getting up from a fall and going round the supermarket Quizzes about preparing for TKR surgery and what to expect *“Traffic light system”* checklist about complications Checklist about home preparations
Concerns about receiving information about the TKR surgical procedure and/or seeing graphic details of TKR surgery	Desire to understand what happens during the TKR surgical procedure	Animation of the TKR surgical procedure, which does not show any graphic details Video of the TKR surgical procedure
Concerns about finding out about *“horror stories”* of TKR surgery Concerns about making comparisons with other patients’ experiences of TKR surgery	Desire to find out about other patients’ experiences of TKR surgery	UK-only moderated online discussion group

*N/A*, not applicable; *TKR*, total knee replacement; *UK*, United Kingdom; *VKS*, Virtual Knee School

**Table 5:** Overview of barriers and facilitators to engagement with a pre-operative total knee replacement exercise program

Barriers to the target behaviour	Facilitators to the target behaviour	VKS design features that could address the barrier and/or facilitator
Knee signs and symptoms	N/A	Non-weight-bearing exercises
Fear that exercising will cause further knee damage	N/A	Reassurance that it is appropriate to exercise with severe knee arthritis
Being busy with other commitments/distractions and forgetting to exercise	N/A	Optional automated email reminders prompting users to perform exercises, with flexible timing Suggestions about setting exercise reminders e.g. on a mobile phone Link between exercise email reminders and personal exercise diary Suggestions about how to integrate exercise into daily routines
Other health issues	N/A	None discussed
Lack of access to specific equipment or facilities	N/A	Exercises that do not require any non-household equipment
Lack of guidance on performing a pre-operative exercise program	N/A	Animations of people demonstrating how to perform exercises Videos of people demonstrating how to perform exercises, with audio explanations of the exercises Tips from peers
Dislike of certain exercise types or delivery modes	Preferences for certain exercise types or delivery modes	Explanations about the benefits of specific exercises/reasons for specific exercises
Setting exercise goals and not meeting them	Setting exercise goals, reviewing exercise goals and receiving feedback about exercise goals	Optional goal setting, review and feedback feature Guidance on how to set realistic goals Goal setting recording sheets that users can download and print out
Short length of time between being listed for TKR surgery and undergoing TKR surgery	Long length of time between being listed for TKR surgery and undergoing TKR surgery	None discussed
N/A	Encouragement from and accountability to health professionals	Exercise diary reviewed health professionals
N/A	Monitoring exercise completion	Private online personal exercise diary Exercise diary that users can download and print out Quick simple exercise recording Use of emojis when recording exercise completion
N/A	Peer support	UK-only moderated online discussion group
N/A	Beliefs about the benefits of pre-operative exercise, including on post-operative recovery, and a sense of personal responsibility for own recovery	Explanations about the benefits of specific exercises/reasons for specific exercises
N/A	Determined personality	This facilitator cannot be directly addressed through the VKS itself

*N/A*, not applicable; *TKR*, total knee replacement; *UK*, United Kingdom; *VKS*, Virtual Knee School

**Table 6:** Overview of barriers and facilitators to engagement with making pre-operative healthy lifestyle changes

Barriers to the target behaviour	Facilitators to the target behaviour	VKS design features that could address the barrier and/or facilitator
**Healthy lifestyle change: Increase physical activity** ^a^		
Knee signs and symptoms	N/A	Non-weight-bearing exercises Guidance on how to pace activities Videos of real people demonstrating how to use walking aids
Concerns about monitoring physical activity	Monitoring physical activity	Physical activity tracker Physical activity recording sheets that users can download and print out Quick simple recording Signposting to fitness tracking apps
**Healthy lifestyle change: Improve weight management and diet**		
Lack of guidance on weight management	N/A	Guidance on weight management strategies Signposting to credible websites that provide general weight management advice
Other health issues or lifestyle choices	N/A	Signposting to credible websites that provide weight management advice that accounts for other health issues or lifestyle choices
Difficulty adhering to diets due to a tendency to overeat	N/A	None discussed
N/A	Beliefs about the benefits of healthy eating and weight management, including on post-operative recovery	Explanations about the benefits of/reasons for healthy eating and weight management
N/A	Monitoring eating habits	Eating habit tracker
N/A	Consultant reprimand/encouragement	None discussed
**Healthy lifestyle change: Reduce alcohol consumption**		
Going on holiday pre-operatively	N/A	None discussed

*N/A*, not applicable; *VKS*, Virtual Knee School

^a^ Barriers and facilitators linked to engaging with a pre-operative exercise program are not included in this table because they are provided in Table 5

## Discussion

This qualitative descriptive study provides novel insights into patients’ perspectives of potential barriers and facilitators to engagement with pre-operative TKR education and prehabilitation digital interventions. A diverse range of barriers and facilitators were identified, many of which depended on participants’ individual circumstances and preferences. Others relate to the pre-operative context. Participants’ perspectives of digital features also appeared to be closely linked to their individual circumstances/preferences and the pre-operative context. These findings highlight the importance of ensuring that the pre-operative TKR digital interventions account for individual differences and are tailored to the pre-operative TKR context.

Participants’ perspectives of barriers, facilitators and digital features were highly varied and often directly opposing. Maximizing the flexibility of digital interventions is vital to account for such differing needs and preferences. Participants in this study suggested digital interventions should include a choice of flexible features, such as optional email reminders sent at user-specified times. Offering choices enables users to self-tailor the intervention, increasing their autonomy [24]. This may enhance users’ intrinsic motivation to engage with the intervention [24, 60], but offering too many choices risks being overwhelming and off-putting for users [61]. Individual differences can also be addressed through computer-tailoring, which involves using computer algorithms to tailor the intervention content/delivery to a user’s individual characteristics [62, 63]. Web-based interventions that employ computer-tailoring may have greater effects on health outcomes than those that do not [64]; however, there are uncertainties about the optimal computer-tailoring strategies and it is important to avoid unnecessarily complex computer-tailoring due to the substantial time/resources it requires [61].

This study identified a wide range of factors linked to the pre-operative context that may affect patient engagement with pre-operative TKR education and prehabilitation digital interventions. Patients’ beliefs about the benefits of pre-operative exercise and healthy lifestyle changes on their post-operative recovery appeared to be a key facilitator to engagement with prehabilitation. This corresponds with the suggestion that the pre-operative phase presents a *‘teachable moment’* – a life transition/event that can motivate an individual to make healthy lifestyle changes (65: p.1, 66: p.156). A range of features that could help to capitalize on patients’ willingness to make pre-operative healthy lifestyle changes were identified in this study, such as providing explanations about the benefits of specific healthy lifestyle changes on TKR outcomes. Social factors also appeared to have an important influence in the pre-operative context, with both professional and peer support appearing to facilitate engagement with pre-operative TKR education and prehabilitation. Conversely, concerns about making comparisons with other patients and hearing negative experiences of TKR surgery were identified as barriers to engagement with pre-operative TKR education. These findings suggest that online TKR discussion groups could be valuable but may need to be moderated to help ensure that inappropriately negative views are not shared.

Many of the barriers and facilitators identified in this study correspond with those identified in previous research. For example, previous studies have highlighted that some patients are reluctant to watch the TKR surgical procedure [26, 67] and patients’ knee signs/symptoms are an important barrier to physical activity [27, 30, 68]. This study extends these findings by highlighting potential approaches for addressing specific barriers and facilitators, such as providing an animation of TKR surgery and including non-weight bearing exercises in prehabilitation programs. This study’s findings also correspond with and expand on previous studies exploring orthopedic patients’ perspectives of digital technologies. For example, a qualitative study by Robinson et al. [17] suggested orthopaedic digital interventions should include features that are user-centered and customizable, aligning with this study’s findings about accounting for individual differences. This study’s findings also suggested that digital features should be quick and simple to use.

### Limitations

This study had a number of limitations, particularly regarding the sample diversity. The diversity was not reflective of UK patients, especially in large urban units. Participants varied in characteristics such as age, gender and educational level, but only one participant was unconfident in using the Internet and none had a disability/health condition that affected their ability to use a website. Barriers and facilitators related to low digital literacy and accessibility may therefore have been overlooked. Furthermore, all participants were White British. Rates of TKR vary between ethnic groups [69] and qualitative research has identified disparities in Black and White patients’ perspectives of post-TKR rehabilitation [70]. Correspondingly, patients from different ethnic groups may face different barriers and facilitators to engagement with pre-operative TKR education and prehabilitation. In addition, only patients able to communicate in English were eligible, despite around 2% of the population in England and Wales not being able to speak English well or at all [71]. This an important limitation as patients unable to communicate in English are likely to face additional barriers to accessing TKR care.

Another limitation of the sample was that only one participant was awaiting her first TKR. A further four participants were awaiting TKR surgery on their second knee, but their perspectives are likely to have been influenced by their previous TKR. Allocating participants to focus groups based on their experience of TKR may have provided more insights into whether the perspectives of patients who were awaiting TKR and those who had undergone TKR differed but was not undertaken due to the pragmatic considerations highlighted in the methods.

Due to COVID-19-related restrictions, all the recruitment was undertaken using community approaches such as social media. As well as limiting the sample diversity, this may have led to the recruitment of individuals who were particularly interested in pre-operative TKR education and prehabilitation and/or digital health, increasing the risk of self-selection bias [72]. To address these issues in future research, it would be valuable to recruit patients from NHS clinics and include additional purposive selection criteria such as ethnicity. These considerations were addressed in the final phase of the VKS project [37]. COVID-19-related restrictions also meant that all the data collection was performed online. Whilst research comparing face-to-face and online focus groups is not entirely consistent, some studies have suggested that the interactivity and depth of data obtained is greater with face-to-face groups [73]. As discussed below, there were some interactivity issues in this study. However, the discussions still generated in-depth data, with participants providing detailed insights into their experiences and perspectives.

Another potential limitation of this study is that the focus groups were facilitated by a single researcher. Lander et al. [74] highlight that involving a co-facilitator (moderator) is not essential for small focus groups but can help to maintain lively interactions. In this study, interactions in the first focus group were initially limited, but that appeared to be due to participants using the online *‘Raise hand’* function whenever they wanted to speak. The facilitator addressed this by emphasizing that they could speak without using the *‘Raise hand’* function, although they were still welcome to use it if they had something to say and felt they were not getting a chance to speak. Subsequent interactions were much livelier despite the online format and having a single facilitator. Interactions in the first and third focus groups were also disrupted due to technical issues and one participant not joining when expected. Having an additional facilitator would have been particularly valuable for resolving those issues.

As highlighted in the methods, the sample size was guided by the aim of achieving saturation. Saturation is considered an important concept for improving the quality of qualitative research by some researchers, yet it remains inconsistently defined and controversial [45–47]. Braun and Clarke [45] have questioned whether saturation is consistent with the principles of reflexive thematic analysis but acknowledge the concept of saturation may be appropriate in some circumstances, such as studies involving a relatively low level of interpretation. In this study, it is possible that using a different approach to determine the sample size, such as information power [75], may have led to further data collection and valuable additional insights.

### Implications for practice and future research

The limitations in pre-operative TKR care provision identified in this study may have implications for clinical practice. The findings suggest improvements may be needed in a range of areas, including provision of pre-operative TKR exercise programs and education on topics such as how to practically prepare for surgery. This study also identified issues related to the timing of pre-operative TKR care delivery, corresponding with a previous UK-based study [76]. Digital interventions may be a promising approach for addressing these issues as they could provide rapid access to pre-operative TKR education and prehabilitation support.

Provision of pre-operative TKR support via digital interventions aligns with the focus on digitally-enabled care in the NHS Long Term Plan [77]. Whilst digital transformation offers many potential benefits, it poses the risk of creating a *‘digital inverse care law’* as patients at risk of digital exclusion often have the highest health support needs (78: ‘Abstract’). Davies et al. [78] highlight that digital exclusion is a complex issue, encompassing inequalities in access to digital connectivity/infrastructure, digital skills/literacy, and patient engagement. The findings of this study support this, with limited experience of using digital tools and reluctance to use digital technologies being identified as barriers to digital engagement. Digital literacy also intersects with health literacy [79]. This is an important consideration as low musculoskeletal health literacy may be associated with worse outcomes following TKR [80]. Given the complexity of digital exclusion, numerous strategies are needed to help address it, such as providing digital skills training programmes, addressing financial barriers to digital technologies, and ensuring that non-digital care options are also available [78, 81].

This study’s findings have informed the development of a VKS prototype as detailed in Anderson et al. [37]. The principles, barriers, facilitators and design features identified could also help guide the development of other TKR digital interventions for use in clinical practice and future research. To address the limitations of this study, future research could explore barriers and facilitators to engagement with pre-operative TKR digital interventions among specific subgroups of patients, such as those who have low digital literacy and/or are from minority ethnic groups. It could also be valuable to conduct face-to-face focus groups on this topic in the future to facilitate interactive and in-depth discussions.

## Conclusions

This qualitative descriptive study identified a wide range of potential barriers and facilitators to engagement with pre-operative TKR digital interventions and design features that could address the barriers and facilitators. The barriers, facilitators and design features relate to two principles that could help to optimize patient engagement with pre-operative TKR education and prehabilitation digital interventions. The first principle highlights the importance of accounting for the impact of individual differences on engagement with digital technologies, pre-operative education and prehabilitation. Maximizing the flexibility of digital interventions through self- and/or computer-tailoring is key to addressing this principle. The second principle suggests that pre-operative TKR education and prehabilitation digital interventions should be tailored to pre-operative contextual features, including physiological/psychological factors, social/occupational factors and limitations in pre-operative TKR care provision. Although most participants in this study felt a pre-operative TKR digital intervention would be valuable, a couple of older participants expressed a general reluctance to use digital technologies. Overall, these findings suggest that pre-operative TKR education and prehabilitation support should be provided via appropriately tailored digital interventions and also available in non-digital formats.

## Electronic supplementary material

Below is the link to the electronic supplementary material.

Supplementary Material 1

Supplementary Material 2

Supplementary Material 3

Supplementary Material 4

Supplementary Material 5: Reviewers’ reports

## Data Availability

The datasets generated and/or analysed during the current study are not publicly available due to the qualitative data including detailed information about the participants’ experiences/perspectives, which could compromise the participants’ anonymity if it was made publicly available, but are available from the corresponding author on reasonable request.

## Abbreviations

DVD: Digital video disc
NHS: National Health Service
PAG: Project Advisory Group
PDF: Portable Document Format
PPI: Patient and Public Involvement
TKR: Total knee replacement
UK: United Kingdom
