# Supplementary material for: “I’m a paper and pencil person”: a qualitative descriptive study of potential barriers and facilitators to engagement with pre-operative total knee replacement education and prehabilitation digital interventions
Source: BMC Musculoskelet Disord. 2025 Jul 4;26:652. doi: 10.1186/s12891-025-08673-1 (PMC12228215; doi:10.1186/s12891-025-08673-1)
Supplement: Supplementary file 1 — Supplementary Material 1 [file 12891_2025_8673_MOESM1_ESM.docx]

**Figure 1 image description**

**Development of a Virtual Knee School, Phase 2**

# Overview

Flowchart summarising the overall Virtual Knee School development project. The flowchart includes boxes labelled as Phase 1a, Phase 1b, Phase 2 and Phase 3 (the intervention planning phases); Phase 4 (the intervention development phase); and Future work (the intervention evaluation and implementation phase).

Each phase is connected to the subsequent phase(s) by a single headed arrow(s). Phase 4 is connected to the Future work by a single headed arrow.

A large arrow at the bottom of the figure demonstrates Patient and Public Involvement continuing throughout all the project phases and future work.

The boxes for each phase summarise the phase design and aim.

# Phase 1a: Mixed methods rapid review

To identify and synthesise recent literature on the content and delivery of pre-operative TKR interventions.

# Phase 1b: Modified Delphi study

To develop recommendations on the content and delivery of pre-operative TKR interventions.

# Phase 2: Qualitative descriptive study

To explore patients’ perspectives of potential barriers and facilitators to engagement with the VKS.

# Phase 3: guiding principles, behavioural analysis, logic model

To use theoretical modelling to guide the design, description and evaluation of the VKS.

# Phase 4: Prototype development, iterative refinement

To develop a prototype VKS and iteratively refine it by evaluating how patients use it and exploring their perspectives of it.
