# Supplementary material for: “I’m a paper and pencil person”: a qualitative descriptive study of potential barriers and facilitators to engagement with pre-operative total knee replacement education and prehabilitation digital interventions
Source: BMC Musculoskelet Disord. 2025 Jul 4;26:652. doi: 10.1186/s12891-025-08673-1 (PMC12228215; doi:10.1186/s12891-025-08673-1)
Supplement: Supplementary file 2 — Supplementary Material 2 [file 12891_2025_8673_MOESM2_ESM.docx]

**Topic Guide, Semi-Structured Interview or Focus Group**

**Development of a Virtual Knee School, Phase 2**

*The following topic guide may be modified during the data collection phase so that themes identified in earlier interviews or focus groups can be explored in later interviews or focus groups. Example prompts are provided; however the specific prompts used may vary. Consent will have been obtained online prior to the interview or focus group.*

# Interview or Focus Group Introduction

*The facilitator(s) should complete all the following actions prior to commencing the interview or focus group*

1. Welcome everyone and complete introductions
2. Review the information provided in the Participant Information Sheet, including:
   - Aim of the study
   - Participants can withdraw at any time
   - Interview or focus group will be recorded with a digital voice recording device
   - Confidentiality
3. Explain the process for the interview or focus group, including:
   - Facilitator(s) will ask prompt questions
   - Facilitator(s) may show digital trigger materials (these will also have been provided in advance via email)
   - Interview or focus group is not a test and there are no right or wrong answers
   - Importance of respecting others’ views
4. Offer the participant(s) an opportunity to ask questions

# Preparing for Surgery Questions

1. Can you tell me about your experiences of receiving information about total knee replacement surgery?

*Prompts may include: Can you tell me about whether you feel you have received enough information? Can you tell me about any additional information you would like to receive? Etc.*

1. Can you tell me about any experiences you have had of doing exercises to help prepare for your total knee replacement surgery?

*Prompts may include: Can you tell me about anything that has prevented you from doing exercises? Can you tell me about anything that has helped you to do exercises? Etc.*

1. Can you tell me about any experiences you have had of making healthy lifestyle changes to help prepare for your total knee replacement surgery?

*Prompts may include: Can you tell me about whether you have tried to lose weight? Can you tell me about anything that has helped you lose weight? Etc.*

1. Can you tell me about anything else that you think is helpful when preparing for total knee replacement surgery?

*Prompts may include: Can you tell me about whether you have spoken to anyone else who has had TKR surgery? Can you tell me about whether you have made, or are planning to make, any changes to your home, such as moving furniture, to help you prepare for your TKR surgery? Etc.*

# Website Questions

1. Can you tell me about any experiences you have had of using websites to help you prepare for your total knee replacement surgery?

*Prompts may include: Can you tell me about any experiences you have had of looking for information about TKR surgery on websites? Have you found any websites particularly helpful and, if so, why? Etc.*

1. Can you tell me about any experiences you have had of using websites for anything else related to your health?

*Prompts may include: Can you tell me about any experiences you have had of using websites to help you carry out exercises? Can you tell me about any experiences you have had of using websites to help you increase your physical activity levels? Etc.*

1. Can you tell me about whether you think there might be any issues with using websites to help prepare for your TKR surgery?

*Prompts may include: Do you have any concerns related to privacy when using websites and, if so, what are your concerns? Can you tell me about whether you think there might be any issues with carrying out an exercise programme that is provided through a website? Etc.*

# Trigger Materials Questions

*More than one set of trigger materials may be used during each interview or focus group.*

1. Can you tell me what you think about using a website to provide <<insert website content>>?

*Prompts may include: Can you tell me what you think about the advice on weight management? Can you tell me whether you would have any problems following the advice? Etc.*

1. Can you tell me what you think about <<insert digital feature/activity/tool>>?

*Prompts may include: Can you tell me about whether you think you would use an activity tracker? Can you tell me about how you would use it? Etc.*

1. Can you tell me about anything that might encourage you to use <<insert website content/digital feature/activity/tool>>?

*Prompts may include: Are there any aspects of the exercise videos you would find particularly helpful? Can you tell me about how you think the exercise instructions could be improved? Etc.*

1. Can you tell me about anything that might prevent you from using <<insert website content/digital feature/activity/tool>>?

*Prompts may include: Do you have any concerns about using an online discussion forum and, if so, what are your concerns? Do you think you would have time to use it? Etc.*

# Final Questions

1. Is there anything else you would like to add?
2. What is your key take-home message from today?

# Interview or Focus Group Closure

*The facilitator(s) should complete all the following actions after the interview or focus group is completed*

1. Thank the participant(s) for taking part in the interview or focus group
2. Remind the participant(s) about confidentiality
3. Ask the participant(s) to complete a travel expenses form if the interview or focus group took place at [location]
