# Supplementary figures and images for: “I’m a paper and pencil person”: a qualitative descriptive study of potential barriers and facilitators to engagement with pre-operative total knee replacement education and prehabilitation digital interventions
Source: BMC Musculoskelet Disord. 2025 Jul 4;26:652. doi: 10.1186/s12891-025-08673-1 (PMC12228215; doi:10.1186/s12891-025-08673-1)

**Coding Example**

**Development of a Virtual Knee School, Phase 2**


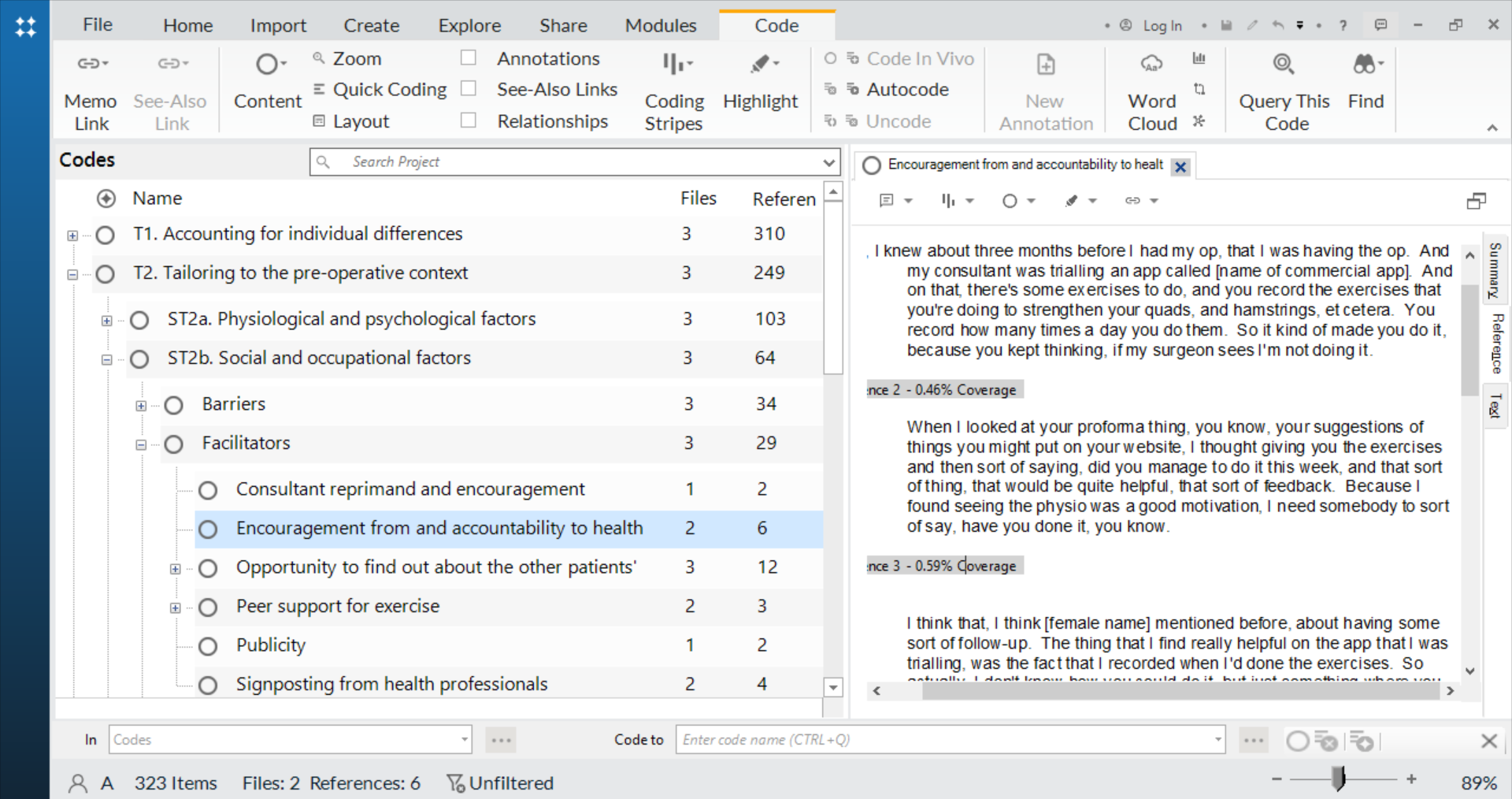

Supplement: Supplementary file 3 — Supplementary Material 3 [file 12891_2025_8673_MOESM3_ESM.docx]
