# Supplementary material for: “I’m a paper and pencil person”: a qualitative descriptive study of potential barriers and facilitators to engagement with pre-operative total knee replacement education and prehabilitation digital interventions
Source: BMC Musculoskelet Disord. 2025 Jul 4;26:652. doi: 10.1186/s12891-025-08673-1 (PMC12228215; doi:10.1186/s12891-025-08673-1)
Supplement: Supplementary file 4 — Supplementary Material 4 [file 12891_2025_8673_MOESM4_ESM.docx]

**Patient Flow Chart**

**Development of a Virtual Knee School, Phase 2**

**Contacts received (n=28)**

Phase 1b (n=8)

Twitter (n=1)

Facebook (n=15)

Word of mouth (n=2)

Unknown (n=2)

**Not screened (n=5)**

Not comfortable participating in a telephone/online discussion (n=1)

No response following provision of the Participant Information Sheet (n=4)

**Screened (n=23)**

**Excluded (n=6)**

Not meeting eligibility criteria (n=1)

Not meeting purposive selection criteria (n=4)

Declined participation (n=1)

**Invited to participate (n=17)**

**Excluded (n=2)**

Did not follow-up on the researcher’s invitation for them to participate (n=2)

**Consented (n=15)**

**Excluded (n=1)**

Unable to join final focus group due to health problems (n=1)

**Participated in a focus group (n=14)**
