# Supplementary material for: “I’m a paper and pencil person”: a qualitative descriptive study of potential barriers and facilitators to engagement with pre-operative total knee replacement education and prehabilitation digital interventions
Source: BMC Musculoskelet Disord. 2025 Jul 4;26:652. doi: 10.1186/s12891-025-08673-1 (PMC12228215; doi:10.1186/s12891-025-08673-1)
Supplement: Supplementary file 5 — Supplementary Material 5: Reviewers’ reports [file 12891_2025_8673_MOESM5_ESM.zip › af87cc7c-f6d8-4382-996a-a8f9367995ba Reviewer reports round 1.docx]

Manuscript title: **“I’m a paper and pencil person”: a qualitative descriptive study of potential barriers and facilitators to engagement with pre-operative total knee replacement education and prehabilitation digital interventions**

First round of peer review

**Reviewer 1**

**Feedback for the author(s):**

Thank you for the opportunity to review this thorough piece of work.

Clear abstract.

Background: Good summary of existing literature. Maybe overstates the novelty of virtual knee school – maybe consider removing word ‘novel’ it is hard to see what is novel about this compared to ones used in many other centres; albeit it seems comprehensive and useful.

Methods – state what phases of MRC model were used or provide figure to illustrate how your study met MRC stages Justification for only including English speakers – how does this match with population receiving TKR?

L191 – consider use of word data saturation – loaded / you need to carefully define what you mean by it against one of the many examples e.g. code or meaning saturation, thematic saturation?– see article by Sebele-Mpofu etc Sebele-Mpofu, Cogent Social Sciences (2020), 6: 1838706https://doi.org/10.1080/23311886.2020.1838706© 2020

Page 8 L 191… consider instead saying no new themes emerged which is clearer and potentially more accurate

Data Analysis – be more succinct – over explained.

Results

Very light on direct quotations – need more to show data derived from the transcripts

Discussion

More needed on digital and health literacy

**Reviewer 2**

**Feedback for the author(s):**

Manuscript title: “I’m a paper and pencil person”: a qualitative descriptive study of potential barriers and facilitators to engagement with pre-operative total knee replacement education and prehabilitation digital interventions.

Thank you for inviting me to review the above manuscript. This qualitative research is on potential barriers and facilitators to engagement in pre-operative total knee replacement education and prehabilitation interventions. The authors used reflexive thematic analysis to conduct their study of 14 patients either listed for Total Knee Replacement (TKR) or had undergone TKR surgery. There are some strengths within the article, in particular the authors have documented their positionality, and it has potential. However, the manuscript requires some major revisions. There is a lack of clarity on the study itself and the qualitative analysis and the Patient and Public Involvement (PPI) requires further detail. I hope my recommendations will promote clarity and quality of the manuscript and continued transparency.

Title

1. I like the use of quotes in titles and the current quote drew me to review the manuscript as it suggested a need for paper information which is a contrast on how we are going with information currently (e.g. more frequently digital). However, this finding was only found with a couple of participants in the study with the majority engaging with digital technology. It implies negative perceptions to digital aspects which wasn’t always the case from the findings. It does not capture the whole study. Can the authors justify the use of this quote.

Abstract

2. Please check that abbreviations can be used within the abstract. I could not locate any information from the guidelines.

3. ‘Remote’ focus groups require some further detail. I note this refers to online/video conferencing, however some readers may not understand this term. Perhaps use online or video conferencing focus groups? I note that one participant joined by telephone – but most video conferencing approaches provide this option and can be explained in more detail in the methods section of the paper. Alternatively, if the authors want to keep ‘remote’ this requires further explanation in the method section on what this involves.

4. ‘Three remote focus groups were conducted to explore their perspectives of barriers/facilitators to …barriers/facilitators.’ Please review this sentence as it is long and confusing. I would suggest replacing ‘their’ with ‘the patients.’

5. Unsure why participants who were awaiting or had undergone TKR were combined in the focus groups? Please see further detail in point 24.

6. I would remove ‘intersecting’ from the results. This raised queries for me straight away if there had been enough review of the themes. I would suggest that the two theme titles are also listed and remove subthemes. For example, ‘Two themes were developed: 1) …(brief description). 2) …(brief description) to quickly signpost the reader to your main findings. The subthemes will be described in the brief descriptions.

7. Although I note the authors are within the word limit – the theme descriptions need to be reduced considerably and further detail can be given to the conclusion – see point 9.

8. The abstract states that the aim of the study is to explore potential barriers/facilitators to engagement of the Virtual Knee School (VKS) – It is my understanding that the VKS is not developed yet. Therefore, this study is more about informing the VKS? Out of interest how was the VKS name determined – were patients involved?

9. The conclusion would benefit from including how the findings will be used and future directions.

10. The authors mention that PPI is involved, and I would suggest that this is also covered in the methods section (however, please see later comments that require further clarity).

Background

11. The background requires more detail about knee osteoarthritis and TKR. There is much literature around why TKR is needed. The authors seem to focus on overweight and obesity on Line 79 but there are many other factors that contribute to resulting in TKR. E.g. It is more commonly noted as a degenerative condition that causes various issues (pain etc). TKR is also more common in older people – wear and tear aspect. I also noted that the focus group question 3 prompt focused on losing weight. Do the authors have a particular focus on overweight patients considering the introduction and question? For example, the prompt would not be applicable for patients who are not overweight, and the introduction does not cover the various factors of end-stage osteoarthritis. If so, this needs to be mentioned.

12. Further UK context would also be beneficial – e.g. how many people are affected by knee osteoarthritis and how many a year undergo TKR for example.

13. Why is there a focus just on TKR? Some patients are also eligible for partial knee replacements (PKR) that is also standard practice? The authors need to justify/explain why there is a particular focus on TKR and why PKR is not included.

14. The gap in the literature is nicely addressed however, further detail on why identifying barriers/facilitators to engagement is important.

Method Design

15. My confusion was further enhanced in this section. I note that this study is part of a wider study. I would recommend that a figure/diagram is used in the design section to show what has been done, where this study sits and what the future involves. It is just briefly mentioned that this study is the second of three intervention planning phases without further description. What do the other stages involve providing the reader some context.

Registration, ethical approval and reporting

16. It is not clear that the approvals cover this study. Perhaps a statement that this study is covered under the wider project.

Research team and oversight

17. It is such a pleasure to see that the authors have covered the team composition and positionality.

18. Focus groups include a co-facilitator to help the facilitator with the process and take notes. Why was a co-facilitator not used? The lead author however also covers the reflection process undertaken. Were there any difficulties for the lead author in the focus groups? If so, this could be a nice reflection in the discussion.

19. Page 7 lines 163-169. Great to see the authors highlight the Project Advisory Group (PAG) and PPI in the project, this paragraph just needs some clarity. It is a bit confusing with the jump of this study back to the wider VKS study here. I am unsure what the role of the PAG and PPI was in this particular study? This needs to be highlighted and promoted and I would recommend the authors provide this section with its own header titled PPI or expertise involved. Further detail on the PAG and PPI involvement is needed and how this directed the study. The current description is broader. For example, I would imagine the PAG must have reviewed this study as it is part of a whole? Were the patient representatives’ people who have lived experience of knee replacement? What the involvement included e.g. reviewing study questions, patient information sheets etc. PPI should be shown throughout the whole research process.

Participants

20. How was the recruitment strategy decided upon?

21. Why was the timeframe of two years for having undergone a TKR decided?

22. If the PAG and PPI were involved in decisions for point 21 and 22, this needs to be highlighted.

23. Sample size – stating that this was guided by data saturation is not consistent with Braun and Clark (2021) reflexive thematic analysis e.g. https://doi.org/10.1080/2159676X.2019.1704846. Similarly, there are guidelines to focus groups – it is recommended to have a least two focus groups with between 4-8 participants. I note the authors have used COREQ that covers data saturation. The authors could address this in the discussion.

24. Why have the authors mixed the participants instead of separating them into pre TKR and post TKR? The authors need to justify why they combined. If this is a limitation – it should be mentioned.

Data collection

25. Page 9 lines 231-232 – Two PAG PPI members were invited to review the Topic Guide but did not suggest any changes.’ Can the authors provide further details. Was a meeting held with them? It is unclear how this review was conducted and if there were any limitations to the process.

26. Page 10 Lines 245-245 ‘The lead researcher recorded field notes during and/or shortly after each focus group.’ Can the authors elaborate why they did this and did this assist with their analysis?

27. Focus groups are well known for their drop-out rates – how did the authors prepare for this?

Data analysis

28. Page 11 lines 279-282 – This paragraph confused me. Are the findings that are reported in this study just focused on the focus groups or does the findings include Phase 1 of the VKS? Again, this jump back to the wider project is very confusing.

Trustworthiness and reflexivity Table 1

29. The mention of focus groups under credibility is also missing that focus groups can enhance discussion through the group interaction of participants sharing similar or different views.

30. How was rapport established?

31. The table again mentions the findings are incorporated with subsequent phases of VKS – Again is this more for the discussion and next steps or did this occur in this study?

32. As outlined in point 26 – how did the reflexive journal help?

33. Maximum variation purposive sampling was used to select a diverse range of participants – the result though was not at times diverse in some areas e.g. only White British participants – noted this is mentioned in the limitations but what went wrong with this approach? Further detail is required and what would the authors recommend in future.

Page 12

34. Were the participants aware of the researcher’s background? If so, what implications could this have?

Results

35. Please outline in the manuscript why 6 participants were not included – note this is mentioned in the patient flow chart, but detail in this section would be helpful. As the PIS is the only abbreviation in the flow chart, I would spell it out in the box rather than just have it on its own in the key.

36. There is a lack of detail on the focus group composition in the results. – e.g., the mix of awaiting and having TKR etc.

Thematic analysis overview

37. Page 13 lines 318-320 – The findings suggested…digital interventions’ is more for the discussion.

38. I found the results very hard to follow as the themes and sub themes are repetitive and cross over. The constant reference of barriers/facilitators is also repetitive.

39. Theme 1 - Is the repetition of engagement with each subtheme necessary? Is theme 1 more about engagement that covers exploring the individual differences? Similarly, the digital aspect can be covered under the pre-operative education as a mode of education. It also comes up again under that section.

40. The prehabilitation section was light and the subthemes could benefit from including a brief description. For example, what is meant by prehabilitation?

41. Theme 2 was just further evidence for what had already been discussed in theme 1.

42. I would recommend the authors to review the themes and rewrite this result section. The narrative is not clear.

43. It is just briefly mentioned that the analysis was discussed with other research team members – what was the frequency of these meetings and how did this help with the analysis? Can be linked in with the reflexive diary and notes etc.

44. Showing a thematic map of the analysis process would help to understand how the themes have been decided upon and the iterative process. The current thematic map is just the overall findings.

Barriers and facilitators table

45. There is a disconnect with the table and the results for me. I’m not sure what the flow is here. Is this table a summary all the barriers/facilitators to take forward applied for the VKS? I note the analysis last step covers the table but its not mentioned how this integrates with the findings.

Discussion

46. The discussion reverts to barriers and facilitators which adds to the confusion of point 46. Perhaps the findings should have a summary of the main barriers and facilitators linked with the analysis and that the barriers and facilitators table shows how this has been adapted for the VKS going forward?

47. The authors cover the main aspects of the discussion, but I would need to review again once the actual study is clearer for me.

Tables

48. Table 2 – The experience of TKR it is listed as pre, post, pre, post and post x2 – unsure of these categories – did some participants have more than one knee operated on?

**Reviewer 3**

**Feedback for the author(s):**

This is a well written piece, it is evident significant time was placed on the writing and editing and proof reading of the submission, thank you for this.

It a nice small scale study, as part of a PhD, which presents some interesting findings, it is important to not overstate the findings of this qualitative study, given the n=14, and the highlighted limitations in the recruitment, the sample demographic and the diversity in pre/post op particulars.

Some revisions i suggest

Line 79: you say "are older adults living with overweight or obesity", I would suggest rewording "living with overweight".

Line 80: are you referring to public patients with regards to waiting times, this should be made clear if so.

Line 244, where you report time of focus groups, I suggest this is a result and not a method and thus should be moved to the results.

Line 305: should this read Figure and not File?

Line 629: in light of the limitations mentioned in the section prior to this, using words like may/could would be better suited to this implications paragraph, so as not to overstate the findings of the study or its subsequent implications.
