# Supplementary material for: “I’m a paper and pencil person”: a qualitative descriptive study of potential barriers and facilitators to engagement with pre-operative total knee replacement education and prehabilitation digital interventions
Source: BMC Musculoskelet Disord. 2025 Jul 4;26:652. doi: 10.1186/s12891-025-08673-1 (PMC12228215; doi:10.1186/s12891-025-08673-1)
Supplement: Supplementary file 5 — Supplementary Material 5: Reviewers’ reports [file 12891_2025_8673_MOESM5_ESM.zip › af87cc7c-f6d8-4382-996a-a8f9367995ba Reviewer reports round 2.docx]

Manuscript title: **“I’m a paper and pencil person”: a qualitative descriptive study of potential barriers and facilitators to engagement with pre-operative total knee replacement education and prehabilitation digital interventions**

Second round of peer review

**Reviewer 1**

**Feedback for the author(s):**

Thank you for the opportunity to review your revised manuscript. I am satisfied that your revision addresses the concerns i raised in my original review (and those of the second reviewer)

**Reviewer 2**

**Feedback for the author(s):**

"PEER REVIEWER ASSESSMENTS:

OBJECTIVE - Full research articles: is there a clear objective that addresses one or several testable research questions? (Brief or other article types: is there a clear objective?)

Yes - there is a clear objective

DESIGN - Is the current approach (including controls and analysis protocols) appropriate for the objective?

Yes - the approach is appropriate

EXECUTION - Are the experiments and analyses performed with sufficient technical rigor to allow confidence in the results?

Yes - experiments and analyses were performed appropriately

STATISTICS - Is the use of statistics in the manuscript appropriate?

Yes - appropriate statistical analyses have been used in the study

INTERPRETATION - Is the current interpretation/discussion of the results reasonable and not overstated?

Yes - the author's interpretation is reasonable

OVERALL MANUSCRIPT POTENTIAL - Has the author addressed your concerns sufficiently for you to now recommend the work as a technically sound contribution? If not, can further revisions be made to make the work technically sound?

Yes - current version is technically sound

PEER REVIEWER COMMENTS:

GENERAL COMMENTS: The reviewers of the previous assessment highlighted a number of important points. Overall, this has resulted in a comprehensive re-writing of the paper. The result is a robust study reported clearly and justified throughout.

The authors have comprehensively addressed the points raised by the two reviewers. The response to Reviewer 1’s comments on MRC framework positioning, saturation, and addition of the paragraph on digital health literacy were particularly clear. The justification for not using the term ‘emerged’ (as suggested by Reviewer 1) and using the term ‘intersecting’ (as suggested by Reviewer 2) is justified from a methodological perspective and is reasonable. This is also clearly explained in the revised paper. Reviewer 2’s comment on sampling people awaiting as well as those who had undergone knee replacement was also addressed in the paper and explored further within the limitations section. The authors have offered more information on the context of the study, both setting and population, to address Reviewer 2’s concern appropriately. A clearer description of the Project Advisory Group and PPI input has also been offered and weaved within the methods (thereby addressing an important point raised by Reviewer 2). Finally, Reviewer 3’s minor points have been comprehensively addressed within the revision.

I am confident that that authors have addressed all points appropriately, the changes are reflective in the paper, and I have no further comments to raise. The addition of the supplementary files and supporting images/figures provide important context which is incredibly helpful. The paper has been robustly reported, following methodological approaches of Braun and Clarke. The Discussion remains pertinent and appropriately interprets the results from this study, within the context of previous understanding and literature. I have nothing to add after my additional critique.

In summary, I believe this to be a robust piece of work which offers important insights into the topic and fully support its acceptance for publication.

ADDITIONAL REQUESTS/SUGGESTIONS:

Nothing additional"

**Reviewer 3**

**Feedback for the author(s):**

Manuscript title: “I’m a paper and pencil person”: a qualitative descriptive study of potential barriers and facilitators to engagement with pre-operative total knee replacement education and prehabilitation digital interventions.

Submission ID: af87cc7c-f6d8-4382-996a-a8f9367995ba

Thank you to the authors for revising the manuscript and for taking some of the previous recommendations on board. The manuscript is much improved with its transparency. However, there are still a number of queries/points that need to be addressed.

Abstract

1. I note that my previous recommendation to include Patient and Public Involvement (PPI) in the methods of the abstract has not been done nor addressed why it has not been included in the authors response – (Previous point 10.) The authors response to this point is in reference to my later point of PPI in the methods in the main paper. I recommend the authors add PPI to the abstract method.

Methods

Design

2. Thanks to the authors for linking in their publication in regards to my previous comment (Previous Point 15). However, it is important to make it easy for the reader and some may not have the time to explore the other publication. As the paper stands, it is not clear how this study fits and what the other phases are. It should not be expected for the reader to look up the other paper. It is also a missed opportunity for promoting where this study fits in the overall picture and the work that has been done in this area. I have looked at this paper the authors have referenced and note this is a great diagram. I would again recommend the authors provide a more succinct/basic diagram of where the study sits and refer to their publication for further information. The sentence on Lines 160-161 could instead state: The overall VKS development project and where this study fits is shown in Figure X, Phase 2. This is a mixed…. (brief sentence). Further detail is reported in Anderson et al. (39). Alternatively, the authors could include a Table briefly describing the phases of the overall study to provide the background detail and refer to the reference for further detail.

Project Advisory Group and Patient and Public Involvement

3. Please amend the title “Project Advisory Group and Patient and Public Involvement’ to just PPI. Project advisory groups are one approach of PPI, as are using PPI representatives (although I am not entirely sure who the PPI representatives are – see later point).

4. Lines 199-200 ‘Two PPI representatives….which included the findings of this study’ please add ‘preliminary’ before findings.

Participants

5. Lines 203-204 – Project Advisory Group can be amended to PAG as this has already been abbreviated.

6. Line 206-207 ‘Phase 1 of the VKS project’ and line 217 – ‘Phases 1 and 4.’ These statements highlight the need to have some background information on the overall study. Please refer to point 2.

7. Whilst I agree on confidentiality, it is appropriate to advise if your PPI representatives have lived experience of the condition you are exploring. Is this not the case? If your PPI representatives do have lived experience of knee replacement/knee osteoarthritis, I would mention this. It is important to be transparent and include the background of the representatives.

8. Line 289 – ‘Two PAG PPI members’ the authors need to be consistent with the terms they use, at other times in the manuscript these members are referred to as PPI representatives. It is important to be consistent with terminology.

Data collection

9. Line 257 ‘…eConsent and Questionnaire..’ what did the questionnaire involve or is this just collecting demographic information? Further detail is required.

10. Lines 266-268 ‘No co-facilitators were involved due to the small size of the focus groups and to ensure that participants could be offered maximum flexibility with the focus group scheduling.’ Depending on the guidelines used typical focus group can consist of e.g. 6-8 participants. Adding how you define the number of participants would help to be clear on why the authors are stating these are small groups.

11. The authors response to my previous review (Previous Point 25), Lines 289-292: ‘Two PAG PPI members were invited to review…timeline’ I would recommend rewording this sentence as it seems to contradict. The first sentence states no feedback, and the second states feedback was via email. This could be amended to ‘Two PAG PPI members were invited via email to review the Topic Guide and no changes were made from their reviews.’ Again, some background on who these individuals were and their expertise is needed.

12. With regards to my previous comment (Previous Point 27) on focus groups and drop-out rates, the authors have stated that rapport was built on telephone contact discussing the study that helped minimise the risk of dropout. This is standard practice for any study to discuss the project with potential participants. Was there anything in particular during these discussions that helped with the rapport building that minimised drop out risk? If so, this would be interesting for other researchers to learn from

13. Lines 300-301 ‘The trigger materials were emailed to the participants prior to their focus group and shared on screen during each focus group.’ How was the screen sharing dealt with the participants on the telephone? Were the participants on the telephone directed to review the material emailed to them at this point?

Data analysis

14. Regarding my previous review (Previous Point 23), whilst the authors state ‘We are aware that Braun and Clark (2021) have questioned the use of saturation in reflexive thematic analysis, although they state ‘Our point here is not that data saturation is never valid and never a useful concept’ please note that Braun and Clark refer to this sentence in regards to 'some forms of thematic analysis' and 'is best avoided' in reflexive thematic analysis. I am unsure what the authors mean by low level interpretation and how this fits with reflexive thematic analysis?

Results

Participants

1. Lines 377-378 – 28 patients contacted the lead researcher and 23 were screened – what happened to the other 5? Was it a case of lost contact?

2. I presume pseudonyms have been used for the participants? Please add a sentence in this section outlining this. It would also be worth explaining the confidence level (provided by the participant? Was this part of the questionnaire?) and age are also listed with the names.

Thematic analysis overview

3. I am still struggling with the themes. The authors have the detail but the current format lacks clarity. Theme 1’s subthemes - as mentioned in my last review, the repetition of ‘Engagement’ in each of the subtheme title is not needed and could imply whether a thorough analysis has been undertaken. I would suggest the authors amend Theme 1’s title to ‘Accounting for individual differences with engagement needs’ and remove engagement for each of the subtheme titles. With the amended title and the description of the theme, engagement is outlined.

4. Theme 2 ‘Tailoring the pre-operative context’ could benefit from being revised. 2a and 2b subtheme titles could be improved. There is some interesting detail in this theme overall and with some revision it has the opportunity to be clearer. Reviewing the three subthemes there is overlap. The authors highlight in 2a providing alternative strategies, tackling misconceptions, understanding the importance of prehabilitation. 2b includes exercise reminders, feedback from others (healthcare professionals and peers). 2c includes importance of prehabilitation, strategies and timing of the preoperative information. These areas can be further condensed, e.g. timing and tackling misconceptions could go into understanding the importance of prehabilitation information. Exercise reminders could come under alternative strategies. For example, the subthemes could potentially be ‘understanding the importance of the prehabilitation, providing alternative strategies, feedback from others to avoid repetition across subthemes.

5. Thematic maps are often used to show the relation between codes to themes and the process of the interpretation of the research findings. Thematic maps can be included for this reason. Please rename ‘thematic map’ as ‘Key themes and subthemes’ as it does not outline the process or an example of how you came to the final themes.

6. Line 424 ‘a mobile app’ please add ‘application’ as this hasn’t been abbreviated. Same with line 120 ‘smartphone apps.’

7. Lines 425-427 ‘and highlighted …TKR’ could me moved to after the paper and pencil person quote to sum these quotes. E.g. Considering the older demographic of patients undergoing TKR, these extracts highlight that the limited experience of using digital tools is an important barrier for some of the older patients.

8. Line 436 ‘Dorothy and other participants’ unsure why you single out Dorothy as there is no quote. Perhaps amend to some of or the majority of patients….

9. Line 448 ‘ In contrast, participants in the third focus group…’ Please amend ‘third’ to ‘another’ for confidentiality reasons and not linking the data to a particular group.

10. Lines 451-422 ‘Participants’ individual circumstances and preferences strongly influenced their perspectives of barriers and facilitators to engagement with digital technologies.’ Lines 468-469 ‘Participants’ differing circumstances and preferences affected their willingness to engage with pre-operative education.’ Lines: 509-511 ‘As for engagement with digital technologies and pre-operative education, participants’ individual circumstances and preferences affected their perspectives of barriers/facilitators to engagement with prehabilitation.’ I would recommend removing these sentences as they are repetitive and this is covered in the overall theme description.

11. Lines 566-569 could be removed if the theme is revised as outlined in point 15.

Tables

12. Tables 3-6 still are still disconnected for me. Some items are not presented in the findings e.g. ‘family member support?’ How were the VKS design features decided upon - This study, PAG, Research team? Further detail is needed.

13. Table 3 the last facilitator on the first page listed seems more like a barrier? ‘Lack of timely access to adequate pre-operative TKR education and prehabilitation from other sources’.

14. Table 3 - Why also are some of the barriers/facilitators in the VKS design column not discussed ‘none discussed’. Further detail in this ‘barriers and facilitators tables’ section is required.

15. I suggest revising the header to Summaries of the barriers and facilitators.

Discussion

16. Lines 468-769 ‘ These findings suggest that online TKR discussion groups could be valuable but need to be appropriately moderated.’ Please add literature to help back this point.
