# Supplementary material for: “I’m a paper and pencil person”: a qualitative descriptive study of potential barriers and facilitators to engagement with pre-operative total knee replacement education and prehabilitation digital interventions
Source: BMC Musculoskelet Disord. 2025 Jul 4;26:652. doi: 10.1186/s12891-025-08673-1 (PMC12228215; doi:10.1186/s12891-025-08673-1)
Supplement: Supplementary file 5 — Supplementary Material 5: Reviewers’ reports [file 12891_2025_8673_MOESM5_ESM.zip › af87cc7c-f6d8-4382-996a-a8f9367995ba Reviewer reports round 3.docx]

Manuscript title: **“I’m a paper and pencil person”: a qualitative descriptive study of potential barriers and facilitators to engagement with pre-operative total knee replacement education and prehabilitation digital interventions**

Third round of peer review

**Reviewer 1**

**Feedback for the author(s):**

Manuscript title: “I’m a paper and pencil person”: a qualitative descriptive study of potential barriers and facilitators to engagement with pre-operative total knee replacement education and prehabilitation digital interventions.

Submission ID: af87cc7c-f6d8-4382-996a-a8f9367995ba

Thank you to the authors for revising the manuscript. I appreciate that the authors have worked hard, and the manuscript is much improved. However, the lack of clarity on the methodology and the themes, and the disconnect with the tables still stands, noting the authors have decided not to revise. I therefore regret to advise that I cannot accept the manuscript for publication. I will leave the final decision to the Journals Editor. I wish the authors all the best with this research and have provided some further detail on points not addressed (as well as other considerations). I hope this helps the authors understand my decision further.

Abstract

1. Thank you for addressing the PPI aspect in the abstract. I recommend keeping this brief in the abstract. This statement doesn’t capture what they further outline their involvement included e.g., recruitment strategy and eligibility criteria: ‘Three Patient and Public Involvement Representatives were involved in reviewing the recruitment aspects of the study including participant facing documentation.’ However also noting that the authors also use a PAG including PPI representative – this is not covered here. Perhaps states ‘PPI Representatives were involved in reviewing the recruitment aspects of the study including participant facing documentation and a Project Advisory Group was involved throughout the study.’

Methods

Project Advisory Group and Patient and Public Involvement

2. The title is still not clear as the PAG included PPI representatives. Perhaps this should just be PAG or Expert Involvement?

3. I recommended using ‘preliminary’ due to my suggested amendments to the findings and it is also common that findings may be revised after a peer-review. If you decide to amend the themes in future, I again recommend using ‘preliminary’ here.

Data collection

4. Thanks for providing further detail why a co-facilitator was not used. However, I am still unsure why this is related to a small focus group. Only one of your groups could be considered small as most guidelines state to have between 5-8 participants. If this wasn’t feasible it should just be stated as that.

Data analysis

5. The authors have referenced four papers published in BMC Musculoskeletal Journal [listed below] to justify their use of data saturation.

Moore, A., Wylde, V., Bruce, J. et al. Experiences of recovery and a new care pathway for people with pain after total knee replacement: qualitative research embedded in the STAR trial. BMC Musculoskelet Disord 23, 451 (2022). <https://doi.org/10.1186/s12891-022-05423-5>

Chua, H., Brady, B., Farrugia, M. et al. Implementing early mobilisation after knee or hip arthroplasty to reduce length of stay: a quality improvement study with embedded qualitative component. BMC Musculoskelet Disord 21, 765 (2020). <https://doi.org/10.1186/s12891-020-03780-7>

Tutuhatunewa, E.D., Stevens, M., Dams, O.C.et al. Exploring patient satisfaction afteroperative and nonoperative treatment for midshaft clavicle fractures: a focus group analysis. BMC Musculoskelet Disord 21, 560 (2020). <https://doi.org/10.1186/s12891-020-03557-y>

Watkins, R., Young, G., Western,M. et al. Nobody says to you “come back in six monthsand we’ll see how you’re doing”: a qualitative interview study exploring young adults’experiences of sport-related knee injury. BMCMusculoskelet Disord 21, 419 (2020). <https://doi.org/10.1186/s12891-020-03428-6>

All four articles refer to using Braun and Clarke’s (2006) approach where data saturation has often been used (although this has not been recommended to use with Braun and Clarke) and are not referencing the version of reflexive thematic analysis that the authors are using. Braun and Clarke’s (2006) paper also makes no reference to data saturation and this has led to further papers from them clarifying their approach and outlining reflexive thematic analysis. As mentioned before, Braun and Clarke have published a paper on this subject stating that their disagreement with such attempts to capture data saturation in thematic analysis led to the following paper: To saturate or not to saturate? Questioning data saturation as a useful concept for thematic analysis and sample-size rationales: Qualitative Research in Sport, Exercise and Health: Vol 13 , No 2 - Get Access (tandfonline.com). The authors present the point on data saturation that Braun and Clark have questioned whether saturation is consistent with reflexive thematic analysis. This does not tie in with Braun and Clarkes extensive work on reflexive thematic analysis.

The authors mention going above the requirements of reporting on saturation and should note there are an abundant number of published reviews that state the importance of outlining data saturation when used. Braun and Clark also outline to assessing thematic analysis research quality that it shouldn’t be assumed that saturation applies to reflexive thematic analysis without any explanation or justification.

The authors reference to ‘low level interpretation (i.e., the focus was on ensuring that the findings closely reflected the participants’ own perspectives) has not been adequately covered and does not fit with reflexive thematic analysis. I recommend that the authors read up on reflexive thematic analysis. Interpretation is key with reflexive thematic analysis. Braun and Clarke state that thematic analysis should not be assumed to offer a low level of interpretation compared to other approaches in their quality practice paper. They further state that ‘even TA [thematic analysis] with a descriptive purpose is an interpretive activity undertaken by the researcher…’

These points have highlighted that the authors need to review the literature on reflexive thematic analysis.

Results

6. The authors should explain in the text that 5 participants were not screened especially as this detail is covered in an additional file.

Thematic analysis overview

7. As previously mentioned, the themes for me are not clear noting that the authors have decided not to revise. The phase in reflexive thematic analysis: ‘Refining, defining and naming themes’ outlines ‘a detailed analysis of each theme, determining the ‘story’ of each. It also involves deciding on an informative name.’ The current themes and sub-themes do not provide this for me. For example, why have psychological and physical factors been grouped and social factors is separate? As previously mentioned, there is also too much cross over highlighting a lack of detailed analysis with the data. The authors have some great findings, but the themes have not been presented clearly in my opinion.

Tables

8. The authors should review previous comments to ensure clarity and add further detail on items not addressed in the main paper as this still causes a disconnect for me including areas not covered in the main paper. This needs to be explained within the manuscript.
