# Supplementary material for: “I’m a paper and pencil person”: a qualitative descriptive study of potential barriers and facilitators to engagement with pre-operative total knee replacement education and prehabilitation digital interventions
Source: BMC Musculoskelet Disord. 2025 Jul 4;26:652. doi: 10.1186/s12891-025-08673-1 (PMC12228215; doi:10.1186/s12891-025-08673-1)
Supplement: Supplementary file 5 — Supplementary Material 5: Reviewers’ reports [file 12891_2025_8673_MOESM5_ESM.zip › Response_To_Reviewers_VKS2_BMC_MSKD_1.0_08Mar24.pdf]

Point-by-point response to reviewers

Manuscript details

Title: "“I’m a paper and pencil person”: a qualitative descriptive study of potential barriers and facilitators to engagement with pre-operative total knee replacement education and prehabilitation digital interventions"
Submission ID: af87cc7c-f6d8-4382-996a-a8f9367995ba
Journal: BMC Musculoskeletal Disorders

Editor comments and response

Thank you for submitting your manuscript to BMC Musculoskeletal Disorders. Please accept our sincere apologies for the delay in the review of your manuscript.
After careful evaluation by our expert reviewers, we would like to request further revisions to your manuscript. Please find below the reviewer’s comments for you to review and address accordingly.
The reviewers have requested further clarification on the study design, methodology, patient and public involvement and the discussion.

Many thanks for reviewing our manuscript and the opportunity to submit a revised version. We have responded to all the reviewers’ comments below and revised our manuscript accordingly. We have ensured that our responses and revisions clarify the study, design, methodology, patient and public involvement and the discussion. The page numbers we have included correspond with the clean version of the manuscript.

Reviewer 1 comments and responses

Thank you for the opportunity to review this thorough piece of work.
Clear abstract.
Background: Good summary of existing literature. Maybe overstates the novelty of virtual knee school – maybe consider removing word ‘novel’ it is hard to see what is novel about this compared to ones used in many other centres; albeit it seems comprehensive and useful.

Thank you very much for your encouraging and constructive feedback. We have now removed the word ‘novel’ from the background.

> Lines 137-139: This study was the second phase of a doctoral research project focused on developing a UK-based, pre-operative TKR education and prehabilitation digital intervention, the *'Virtual Knee School'* (VKS) (35).

Methods – state what phases of MRC model were used or provide figure to illustrate how your study met MRC stages.

We have now added details about how this study maps to the MRC framework and provided a reference to a published paper that provides a diagram of the overall VKS development. We submitted this focus group manuscript to *BMC Musculoskeletal Disorders* on the 10th of March 2023, prior to submitting the VKS development paper, so signposting to the VKS development paper in our original manuscript was not possible.

> Lines 155-161: The VKS project maps to the development stage of the MRC framework (37). This highlights that primary research with the intended intervention users can be useful for developing a theoretical understanding of how a proposed intervention may achieve its intended changes (37). The overall VKS project employed a complex mixed methods design, primarily underpinned by pragmatism (38). This study was the second of three intervention planning phases. The overall VKS development project is reported in Anderson et al. (39).

Justification for only including English speakers – how does this match with population receiving TKR?

We have now added justification for only including English speakers to the methods.

> Lines 214-215: Inclusion of patients unable to communicate in English was not possible because translation and interpreting funds were not available.

We have also acknowledged this as a limitation in the discussion. We have provided the percentage of the population in England and Wales who are not English speakers as we are not aware of data reporting the percentage of patients undergoing TKR in England and Wales who are not English speakers.

> Lines 793-797: In addition, only patients able to communicate in English were eligible, despite around 2% of the population in England and Wales not being

> able to speak English well or at all (72). This an important limitation as patients unable to communicate in English are likely to face additional barriers to accessing TKR care.

L191 – consider use of word data saturation – loaded / you need to carefully define what you mean by it against one of the many examples e.g. code or meaning saturation, thematic saturation?– see article by Sebele-Mpofu etc Sebele-Mpofu, Cogent Social Sciences (2020), 6: 1838706https://eur03.safelinks.protection.outlook.com/?url=https%3A%2F%2Fdoi.org%2F10.1080%2F23311886.2020.1838706%25C2%25A9&data=05%7C02%7CA.Anderson%40leeds.ac.uk%7C860b475705a9429ad14208dc387fc05c%7Cbdeaeda8c81d45ce863e5232a535b7cb%7C0%7C0%7C638447368548925687%7CUnknown%7CTWFpbGZsb3d8eyJWIjoiMC4wLjAwMDAiLCJQIjoiV2luMzIiLCJBTiI6Ik1haWwiLCJXVCI6Mn0%3D%7C0%7C%7C%7C&sdata=7iAu4t8Qb35k0D25yhQLZNUsMjMZ54JbkXea2bUc9DE%3D&reserved=0 2020

We appreciate you sharing that helpful article and have now expanded the text on saturation in the methods and discussion sections to incorporate it.

> Lines 229-247: There is no universally accepted approach for determining the sample size in qualitative studies (47, 48). Aiming to achieve saturation is one of the most widely used approaches, with some authors considering it an important measure of quality in qualitative research (49). Saturation may be broadly conceptualized as the point at which further data collection does not provide any new information (50). However, additional qualitative data arguably always provide some new insights, and the concept of saturation is inconsistently defined, with various authors suggesting different types of saturation (47, 49). Empirical tests of saturation typically involve counting codes and rely on a relatively fixed codebook (47, 51). Such tests are incompatible with the fluid coding approach used in reflexive thematic analysis (47). Correspondingly, Braun and Clarke (47) argue that data saturation is not a valid concept for all qualitative studies, particularly those that are highly interpretative. When data saturation is referred to, Braun and Clarke (47) recommend clarifying how it is conceptualized.
>
> This study involved a relatively low level of interpretation. Aiming to achieve saturation was therefore considered an appropriate approach for determining the

> sample size, but counting codes to assess saturation was not. Correspondingly, saturation was assessed subjectively and conceptualized as the point at which additional data collection was considered unlikely to lead to the identification of new themes, which may be considered to align with data or thematic saturation (49, 52).

> Lines 831-839: As highlighted in the methods, the sample size was guided by the aim of achieving saturation. Saturation is considered an important concept for improving the quality of qualitative research by some researchers, yet it remains inconsistently defined and controversial (47-49). Braun and Clarke (47) have questioned whether saturation is consistent with the principles of reflexive thematic analysis but acknowledge the concept of saturation may be appropriate in some circumstances, such as studies involving a relatively low level of interpretation. In this study, it is possible that using a different approach to determine the sample size, such as information power (76), may have led to further data collection and valuable additional insights.

Page 8 L 191… consider instead saying no new themes emerged which is clearer and potentially more accurate

We have used reflexive thematic analysis as described by Braun and Clarke, so do not feel it is appropriate to use the term *'emerged'* as Braun and Clarke are critical of that terminology, as detailed in the quote below from Braun and Clark (2021).

> 'We encourage researchers using reflexive TA to write about theme generation as a creative and active process, one they are central to, and to always avoid claiming that themes emerged.'

Data Analysis – be more succinct – over explained.

We have now amended our main description of the data analysis to be more succinct, although the analysis section is now longer overall due to the extra details requested by reviewer 2.

> Lines 320-339: The lead researcher led the analysis by employing the six phases of reflexive thematic analysis flexibly. This included coding the transcripts using a fluid and largely semantic approach to help ensure that the codes reflected

participants' perspectives. Codes were clustered into candidate themes/subthemes, which were critically reviewed. This led to redevelopment of all the themes/subthemes and collapsing of some themes/subthemes. A key change was collapsing two candidate themes, *'Tailoring to pre-operative phase barriers'* and *'Targeting key facilitators'*, into a single theme, *'Tailoring to the pre-operative phase'*.

Field notes were used to assist the analysis where appropriate, with the summary of the overall focus group discussions being particularly useful for ensuring key points emphasized by participants were considered. QSR International NVivo software (Version 12 and Release 1) was employed to facilitate organization of the data. Additional File 2 provides an example of the theme/subtheme coding structure in the final NVivo file.

In addition to the narrative report, tables were created to summarize the barriers, facilitators and digital features from across both themes. Separate tables were created for each behaviour targeted by the VKS. This approach was based on a previous evidence-, theory- and person-based intervention planning study (60) and ensured that the data were optimally structured for informing the subsequent project phases, which are described in Anderson et al. (39).

Results

Very light on direct quotations – need more to show data derived from the transcripts

We have added further direct quotations throughout the results section.

Lines 444-446: *"I think the one that I mentioned, the one the physio sent me, which was real people, and I think that helps better than doing an animation or, you know, just a stick figure on a piece of paper."* (Sophia, 40–49, very confident, post-TKR)

Lines 462-465: *"So I think something that actually pings up and says, oi, go and do, you know, ten stand and sits, now, to me, is probably more useful than having a notification coming through say in an email, that goes, open an email and it says, you should be doing this now."* (Jacob, 50–59, very confident, post-TKR)

Lines 474-476: *“I think a bit more advice on how to prepare the home for when you came back from hospital, certainly for me, would have been useful if I'd had that before the op.”* (Irene, 50–59, confident, post-TKR)

Lines 484-486: *“I did not want to know at all what they were going to do to me. And anybody who started to tell me, I just switched off, I did not want to know or to see anything, or to read about it or see pictures.”* (Dorothy, 70–79, confident, post-TKR)

Lines 525-528: *“So I was all prepped up ready and then they said hang on, your heartrate’s too low. That’s because of all the physical exercise I do. So I had to slow down my exercise so they were going to go back four months later and have my operation done then.”* (Cameron, 60–69, very confident, post-TKR)

Lines 534-537: *“And some people would say, you know, I can't do this, or I've got that, and if you just referred them to the NHS page, it covers pretty much, basically everything, and all different, you know, eventualities of being vegan, or you know, diabetic, or anything like that.”* (Sophia, 40–49, very confident, post-TKR)

Lines 543-545: *“I’d been going to the gym four or five times a week, and swimming, but then obviously lockdown happened, and I’ve not done as much.”* (Molly, 40–49, very confident, pre-TKR)

Lines 5760577: *“The only one I had was pain and it got so bad that I had to stop [exercising].”* (Luke, 60–69, very confident, pre- and post-TKR)

Lines 598-602: *“I know this might sound really counterintuitive but I said to myself, if I don't do exercise or carry on doing some exercise, when I get to post-op my knee…I might not have any sufficient muscles or anything else around my knee to help me. So I said to myself I've got to just keep it going […].”* (Cameron, 60–69, very confident, post-TKR)

Lines 607-609: *“I was just finding that when I was doing the exercises, I was finding it very, very difficult, but the more I did, the more it loosened up my other joints and I found it very, very helpful.”* (Ivy, 50–59, confident, post-TKR on both knees)

> Lines 628-632: *"Yes, I think so because once you've had the operation, you know you've got to exercise and initially all you're doing all day is exercising and taking tablets. So you know that's an easy routine to get into. I had a timetable as well. But beforehand when life was much more normal and very full, yes, I think reminders would be useful."* (Dorothy, 70–79, confident, post-TKR)
>
> Lines 661-665: *"I had the mother, who had gone through the experience, and there was a friend who goes to a support group that I go to, who had the horror story from hell of her experience. And if I heard it once, I heard it three dozen times, from her, of what had happened to her. So, no, I didn't use Google, because I didn't want to find out more horror stories."* (Olivia, 50–59, very confident, post-TKR)
>
> Lines 687-689: *"It could be good to have a checklist of things you need to get prepared at home before you go in. Because you don't always get the information from your physio or your knee school at the hospital."* (Molly, 40–49, very confident, pre-TKR)
>
> Lines 695-698: *"So when I got my package with, it was a very well prepared booklet thing that I was given, with a color coded card for each aspect of the operation, and exercise, and everything, all in a nice folder I was given. But it wasn't really long enough beforehand, in a way."* (Beatrice, 70–79, confident, pre- and post-TKR)

Discussion

More needed on digital and health literacy

We have now added a paragraph addressing this to the discussion.

> Lines 851-863: Provision of pre-operative TKR support via digital interventions aligns with the focus on digitally-enabled care in the NHS Long Term Plan (78). Whilst digital transformation offers many potential benefits, it poses the risk of creating a *'digital inverse care law'* as patients at risk of digital exclusion often have the highest health support needs (79: 'Abstract'). Davies et al. (79) highlight that digital exclusion is a complex issue, encompassing inequalities in access to digital connectivity/infrastructure, digital skills/literacy, and patient engagement.

> The findings of this study support this, with limited experience of using digital tools and reluctance to use digital technologies being identified as barriers to digital engagement. Digital literacy also intersects with health literacy (80). This is an important consideration as low musculoskeletal health literacy may be associated with worse outcomes following TKR (81). Given the complexity of digital exclusion, numerous strategies are needed to help address it, such as providing digital skills training programmes, addressing financial barriers to digital technologies, and ensuring that non-digital care options are also available (79, 82).

Reviewer 2 comments and responses

Thank you for inviting me to review the above manuscript. This qualitative research is on potential barriers and facilitators to engagement in pre-operative total knee replacement education and prehabilitation interventions. The authors used reflexive thematic analysis to conduct their study of 14 patients either listed for Total Knee Replacement (TKR) or had undergone TKR surgery. There are some strengths within the article, in particular the authors have documented their positionality, and it has potential. However, the manuscript requires some major revisions. There is a lack of clarity on the study itself and the qualitative analysis and the Patient and Public Involvement (PPI) requires further detail. I hope my recommendations will promote clarity and quality of the manuscript and continued transparency.

Many thanks for taking the time to provide detailed feedback about our manuscript.

Title

1. I like the use of quotes in titles and the current quote drew me to review the manuscript as it suggested a need for paper information which is a contrast on how we are going with information currently (e.g. more frequently digital). However, this finding was only found with a couple of participants in the study with the majority engaging with digital technology. It implies negative perceptions to digital aspects which wasn't always the case from the findings. It does not capture the whole study. Can the authors justify the use of this quote.

We are glad to hear that this quote drew you to review the manuscript and agree that the quote reflects the perspective of a minority of participants. We believe this is appropriate as highlighting the perspective of minority groups is vital to ensure that research and clinical practice help to address rather than compound existing health inequities. This is

particularly important in the case of digital exclusion due to the risk of creating a digital inverse care law. We have now expanded on this point in our discussion as suggested by reviewer 1.

> Lines 851-863: Provision of pre-operative TKR support via digital interventions aligns with the focus on digitally-enabled care in the NHS Long Term Plan (78). Whilst digital transformation offers many potential benefits, it poses the risk of creating a 'digital inverse care law' as patients at risk of digital exclusion often have the highest health support needs (79: 'Abstract'). Davies et al. (79) highlight that digital exclusion is a complex issue, encompassing inequalities in access to digital connectivity/infrastructure, digital skills/literacy, and patient engagement. The findings of this study support this, with limited experience of using digital tools and reluctance to use digital technologies being identified as barriers to digital engagement. Digital literacy also intersects with health literacy (80). This is an important consideration as low musculoskeletal health literacy may be associated with worse outcomes following TKR (81). Given the complexity of digital exclusion, numerous strategies are needed to help address it, such as providing digital skills training programmes, addressing financial barriers to digital technologies, and ensuring that non-digital care options are also available (79, 82).

We also agree that this quote does not capture the whole study. However, we do not feel that is an issue as the second part of the title does capture the whole study.

> Title: […] a qualitative descriptive study of potential barriers and facilitators to engagement with pre-operative total knee replacement education and prehabilitation digital interventions

Abstract

2. Please check that abbreviations can be used within the abstract. I could not locate any information from the guidelines.

The BMC Musculoskeletal Disorders submission guidelines state 'Please minimize the use of abbreviations and do not cite references in the abstract.'

We have now written out 'UK' in full so the abstract only includes two abbreviations – TKR (total knee replacement) and VKS (Virtual Knee School).

3. ‘Remote’ focus groups require some further detail. I note this refers to online/video conferencing, however some readers may not understand this term. Perhaps use online or video conferencing focus groups? I note that one participant joined by telephone – but most video conferencing approaches provide this option and can be explained in more detail in the methods section of the paper. Alternatively, if the authors want to keep ‘remote’ this requires further explanation in the method section on what this involves.

We have replaced ‘remote’ with online throughout the manuscript.

> Lines 35-36: Three online focus groups were conducted.
>
> Lines 268-269: Due to COVID-19-related restrictions, the focus groups were held online using Blackboard CollaborateTM, a secure videoconferencing tool.
>
> Lines 813-815: COVID-19-related restrictions also meant that all the data collection was performed online, which may limit the depth of information gained (74).

4. ‘Three remote focus groups were conducted to explore their perspectives of barriers/facilitators to …barriers/facilitators.’ Please review this sentence as it is long and confusing. I would suggest replacing ‘their’ with ‘the patients.’

We have split this sentence into two and replaced ‘their’ with ‘the patients’.

> Lines 35-38: Three online focus groups were conducted. The focus groups explored patients’ perspectives of barriers and facilitators to engagement with the behaviors targeted by the VKS and digital features that could address the barriers and facilitators.

5. Unsure why participants who were awaiting or had undergone TKR were combined in the focus groups? Please see further detail in point 24.

We have now added further justification for this decision to the methods section.

> Lines 258-263: Participants were primarily allocated to the focus groups based on when they were recruited and their availability/timing preferences, rather than

> their experience of TKR. This approach was chosen because ensuring the timing of focus groups is convenient for patients may encourage them to participate (54). Furthermore, not all participants had been recruited at the time of the first and second focus groups as the recruitment overlapped with the data collection.

We have also now highlighted this in the limitations section.

> Lines 799-805: Another limitation of the sample was that only one participant was awaiting her first TKR. A further four participants were awaiting TKR surgery on their second knee, but their perspectives are likely to have been influenced by their previous TKR. Allocating participants to focus groups based on their experience of TKR may have provided more insights into whether the perspectives of patients who were awaiting TKR and those who had undergone TKR differed but was not undertaken due to the pragmatic considerations highlighted in the methods.

6. I would remove 'intersecting' from the results. This raised queries for me straight away if there had been enough review of the themes. I would suggest that the two theme titles are also listed and remove subthemes. For example, 'Two themes were developed: 1) …(brief description). 2) …(brief description) to quickly signpost the reader to your main findings. The subthemes will be described in the brief descriptions.

We believe it is important to include the word *'intersecting'* in our results to show that the themes are connected to each other, as detailed in our thematic map. In their book, '[Thematic analysis: a practical guide'] Braun and Clarke (2021) emphasise that themes need to have a central organising concept, but linkages and relationships between themes and subthemes are appropriate.

We have now updated the text of the abstract results section as suggested.

> Lines 40-50: Two intersecting themes were developed. Theme 1, *'Accounting for individual differences'*, suggests pre-operative TKR digital interventions should account for the impact of individual differences on engagement with digital technologies, pre-operative education and prehabilitation. Most participants felt a pre-operative TKR digital intervention would be valuable; however, a couple of older participants appeared reluctant to use digital technologies. Participants' perspectives of specific digital features and pre-operative TKR education and

> prehabilitation also varied widely. Theme 2, '*Tailoring to the pre-operative context'* highlights the importance of tailoring pre-operative TKR digital interventions to pre-operative contextual features, including physiological/psychological factors, social/occupational factors and limitations in pre-operative TKR care provision. Various digital features that could address these factors were identified.

7. Although I note the authors are within the word limit – the theme descriptions need to be reduced considerably and further detail can be given to the conclusion – see point 9.

We have now amended the abstract results and conclusion sections as suggested and detailed in our responses to points 6 and 9.

8. The abstract states that the aim of the study is to explore potential barriers/facilitators to engagement of the Virtual Knee School (VKS) – It is my understanding that the VKS is not developed yet. Therefore, this study is more about informing the VKS? Out of interest how was the VKS name determined – were patients involved?

We have clarified that the purpose of exploring potential barriers and facilitators to engagement with the VKS was to inform its development.

> Lines 29-33: This study was part of a project focused on developing a novel pre-operative TKR education and prehabilitation digital intervention, the *'Virtual Knee School'* (VKS), and aimed to explore patients' perspectives of potential barriers/facilitators to engagement with the VKS to help inform its development.

The name Virtual Knee School was decided when planning the grant application for this project. Patients were involved in the project planning, although the name *'Virtual Knee School'* was primarily chosen for consistency with pre-operative education provision in the UK, which is typically undertaken at group classes often called *'knee schools'* as detailed in our background section.

> Lines 102-103: Pre-operative TKR support has commonly been delivered via face-to-face group classes, often known as *'knee schools'* (13, 18: p.118).

9. The conclusion would benefit from including how the findings will be used and future directions.

We have now expanded the abstract conclusions section as suggested.

> Lines 51-57: This study's findings suggest pre-operative TKR digital interventions should account for individual differences and be tailored to the pre-operative TKR context. A range of barriers/facilitators and digital features to consider when developing pre-operative TKR digital interventions were identified. Given that some patients are reluctant to use digital technologies, also offering pre-operative TKR support in non-digital formats is essential. This study's findings have been used to inform a VKS prototype and could also be used to inform the development of other pre-operative TKR digital interventions.

10. The authors mention that PPI is involved, and I would suggest that this is also covered in the methods section (however, please see later comments that require further clarity).

We have now added a specific section on the roles of the Project Advisory Group and PPI to the methods.

> Lines 186-200: The VKS project was overseen by a Project Advisory Group (PAG). Full PAG meetings were held approximately every six months throughout the 39-month project. The PAG included an independent chair, a collaborator from a local association of acute NHS trusts, three Patient and Public Involvement (PPI) representatives with diverse lived experiences relevant to the study (one of whom was recruited after this study), the lead researcher (AMA) and three of her supervisors.
>
> Key roles of the PAG in this specific study included discussing the project planning and setting and reviewing success criteria. In addition, the PPI representatives reviewed this study's participant documents, Topic Guide, digital trigger materials (examples of website features shown to participants), and/or plain English summary. This led to various changes, such as increasing the font size on the participant documents, including a pain tracker in the digital trigger materials, and amending the layout and wording of the plain English summary.

> Two PPI representatives were also involved in planning and co-presenting a public dissemination event, which included the findings of this study.

Background

11. The background requires more detail about knee osteoarthritis and TKR. There is much literature around why TKR is needed. The authors seem to focus on overweight and obesity on Line 79 but there are many other factors that contribute to resulting in TKR. E.g. It is more commonly noted as a degenerative condition that causes various issues (pain etc). TKR is also more common in older people – wear and tear aspect. I also noted that the focus group question 3 prompt focused on losing weight. Do the authors have a particular focus on overweight patients considering the introduction and question? For example, the prompt would not be applicable for patients who are not overweight, and the introduction does not cover the various factors of end-stage osteoarthritis. If so, this needs to be mentioned.

We have now added data to justify why we mentioned that most patients undergoing TKR are older adults who are overweight or obese.

> Lines 82-86: Key risk factors for OA include increasing age, previous knee injury, female gender, and being overweight or obese (5). Correspondingly, the average age of patients undergoing TKR is 70 years old, approximately 56% of patients undergoing TKR are female, and approximately 90% are overweight or obese (6, 7).

We have also highlighted that weight management is a component of prehabilitation to further justify why weight management was considered during this study.

> Lines 95-97: A multimodal approach to prehabilitation is generally advocated, which may include various strategies such as exercise programs, weight management, and alcohol reduction (14, 15).

12. Further UK context would also be beneficial – e.g. how many people are affected by knee osteoarthritis and how many a year undergo TKR for example.

We have now added further UK context.

> Lines 77-82: Total knee replacement (TKR) is a common elective orthopaedic procedure, with over 83,000 TKRs undertaken in England in 2022 (1). TKR is usually undertaken to relieve the pain and other disabling symptoms of end-stage knee osteoarthritis (OA) (2). OA is a chronic disease in which the destructive and reparative processes of joint tissues are imbalanced, leading to structural alterations throughout the joint (3). Estimates suggest over 4,538,000 people in the United Kingdom (UK) have OA (4).

13. Why is there a focus just on TKR? Some patients are also eligible for partial knee replacements (PKR) that is also standard practice? The authors need to justify/explain why there is a particular focus on TKR and why PKR is not included.

We have now justified this.

> Lines 139-142: The VKS was developed specifically for patients undergoing TKR because factors such as patient demographics, length of hospital stay, and recovery timescales vary between TKR and other orthopaedic procedures such as partial knee replacement.

14. The gap in the literature is nicely addressed however, further detail on why identifying barriers/facilitators to engagement is important.

We have now added further detail about this.

> Lines 114-120 Rigorously developing digital interventions is vital to address these issues (25). When planning a novel digital intervention, it is important to explore the intended users' perspectives of the behaviors the intervention seeks to change (26). Identifying barriers and facilitators to the target behaviors is particularly valuable to enable features addressing the barriers and facilitators to be incorporated into the intervention (26). This can help to ensure that the intervention being developed is acceptable, engaging and supportive of the desired behaviour changes (26).

Method

Design

15. My confusion was further enhanced in this section. I note that this study is part of a wider study. I would recommend that a figure/diagram is used in the design section to

show what has been done, where this study sits and what the future involves. It is just briefly mentioned that this study is the second of three intervention planning phases without further description. What do the other stages involve providing the reader some context.

We have now signposted to a paper that provides a diagram of the overall VKS development and where this study sits. We submitted this focus group manuscript to *BMC Musculoskeletal Disorders* on the 10th of March 2023, prior to submitting the VKS development paper, so signposting to the VKS development paper in our original manuscript was not possible.

> Lines 160-161: The overall VKS development project is reported in Anderson et al. (39).

Registration, ethical approval and reporting

16. It is not clear that the approvals cover this study. Perhaps a statement that this study is covered under the wider project.

We have now clarified that the approval covers this study.

> Lines 171-172: Ethical approval covering this study was obtained from the Yorkshire and The Humber - Bradford Leeds Research Ethics Committee (20/YH/0095).

Research team and oversight

17. It is such a pleasure to see that the authors have covered the team composition and positionality.

Thank you for this positive feedback.

18. Focus groups include a co-facilitator to help the facilitator with the process and take notes. Why was a co-facilitator not used? The lead author however also covers the reflection process undertaken. Were there any difficulties for the lead author in the focus groups? If so, this could be a nice reflection in the discussion.

There is no definitive guidance on how focus groups should be conducted. We have now added our justification for not involving co-facilitators in the methods.

> Lines 266-268: No co-facilitators were involved due to the small size of the focus groups and to ensure that participants could be offered maximum flexibility with the focus group scheduling.

We have also added reflections on this to the discussion.

> Lines 819-829: Another potential limitation of this study is that the focus groups were facilitated by a single researcher. Lander et al. (75) highlight that involving a co-facilitator (moderator) is not essential for small focus groups but can help to maintain lively interactions. In this study, interactions in the first focus group were initially limited, but that appeared to be due to participants using the online *'Raise hand'* function whenever they wanted to speak. The facilitator addressed this by emphasizing that could speak without using the *'Raise hand'* function, although they were still welcome to use it if they had something to say and felt they were not getting a chance to speak. Subsequent interactions were much livelier despite having a single facilitator. Interactions in the first and third focus groups were also disrupted due to due to technical issues and one participant not joining when expected. Having an additional facilitator would have been particularly valuable for resolving those issues.

19. Page 7 lines 163-169. Great to see the authors highlight the Project Advisory Group (PAG) and PPI in the project, this paragraph just needs some clarity. It is a bit confusing with the jump of this study back to the wider VKS study here. I am unsure what the role of the PAG and PPI was in this particular study? This needs to be highlighted and promoted and I would recommend the authors provide this section with its own header titled PPI or expertise involved. Further detail on the PAG and PPI involvement is needed and how this directed the study. The current description is broader. For example, I would imagine the PAG must have reviewed this study as it is part of a whole? Were the patient representatives' people who have lived experience of knee replacement? What the involvement included e.g. reviewing study questions, patient information sheets etc. PPI should be shown throughout the whole research process.

We have now given this section its own header, added the role of the PAG and PPI in this specific study, and highlighted that the PPI representatives had diverse lived

experiences relevant to the study. We have not added specific details of the PPI representatives' lived experiences for confidentiality reasons.

> Lines 186-200: The VKS project was overseen by a Project Advisory Group (PAG). Full PAG meetings were held approximately every six months throughout the 39-month project. The PAG included an independent chair, a collaborator from a local association of acute NHS trusts, three Patient and Public Involvement (PPI) representatives with diverse lived experiences relevant to the study (one of whom was recruited after this study), the lead researcher (AMA) and three of her supervisors.
>
> Key roles of the PAG in this specific study included discussing the project planning and setting and reviewing success criteria. In addition, the PPI representatives reviewed this study's participant documents, Topic Guide, digital trigger materials (examples of website features shown to participants), and/or plain English summary. This led to various changes, such as increasing the font size on the participant documents, including a pain tracker in the digital trigger materials, and amending the layout and wording of the plain English summary. Two PPI representatives were also involved in planning and co-presenting a public dissemination event, which included the findings of this study.

Participants

20. How was the recruitment strategy decided upon?

We have now added details about this.

> Lines 203-205: The recruitment strategy and eligibility criteria were discussed and finalized with the Project Advisory Group. A pragmatic approach was essential because the study was conducted in the early stages of the COVID-19 pandemic.

21. Why was the timeframe of two years for having undergone a TKR decided?

We have now added details about this.

> Lines 215-219: The timeframe of two years for having undergone a TKR was chosen to ensure participants could remember their pre-operative experiences and is consistent with the approach used in Phases 1 and 4 of the VKS project (39, 44).

22. If the PAG and PPI were involved in decisions for point 21 and 22, this needs to be highlighted.

We have now added details about this.

> Lines 203-205: The recruitment strategy and eligibility criteria were discussed and finalized with the Project Advisory Group. A pragmatic approach was essential because the study was conducted in the early stages of the COVID-19 pandemic.

23. Sample size – stating that this was guided by data saturation is not consistent with Braun and Clark (2021) reflexive thematic analysis e.g. https://eur03.safelinks.protection.outlook.com/?url=https%3A%2F%2Fdoi.org%2F10.1080%2F2159676X.2019.1704846&data=05%7C02%7CA.Anderson%40leeds.ac.uk%7C860b475705a9429ad14208dc387fc05c%7Cbdeaeda8c81d45ce863e5232a535b7cb%7C0%7C0%7C638447368548929773%7CUnknown%7CTWFpbGZsb3d8eyJWIjoiMC4wLjAwMDAiLCJQIjoiV2luMzIiLCJBTiI6Ik1haWwiLCJXVCI6Mn0%3D%7C0%7C%7C%7C&sdata=yPO%2BCrkSJU%2B2%2FN3k8z2dokujzBJxWp9sRE7HAKyiHJo%3D&reserved=0. Similarly, there are guidelines to focus groups – it is recommended to have a least two focus groups with between 4-8 participants. I note the authors have used COREQ that covers data saturation. The authors could address this in the discussion.

We are aware that Braun and Clark (2021) have questioned the use of saturation in reflexive thematic analysis, although they also state *'Our point here is not that data saturation is never valid and never a useful concept.'* To help address this, we have expanded our discussion of saturation in the methods and discussion sections.

> Lines 229-247: There is no universally accepted approach for determining the sample size in qualitative studies (47, 48). Aiming to achieve saturation is one of the most widely used approaches, with some authors considering it an important measure of quality in qualitative research (49). Saturation may be broadly conceptualized as the point at which further data collection does not provide any

new information (50). However, additional qualitative data arguably always provide some new insights, and the concept of saturation is inconsistently defined, with various authors suggesting different types of saturation (47, 49). Empirical tests of saturation typically involve counting codes and rely on a relatively fixed codebook (47, 51). Such tests are incompatible with the fluid coding approach used in reflexive thematic analysis (47). Correspondingly, Braun and Clarke (47) argue that data saturation is not a valid concept for all qualitative studies, particularly those that are highly interpretative. When data saturation is referred to, Braun and Clarke (47) recommend clarifying how it is conceptualized.

This study involved a relatively low level of interpretation. Aiming to achieve saturation was therefore considered an appropriate approach for determining the sample size, but counting codes to assess saturation was not. Correspondingly, saturation was assessed subjectively and conceptualized as the point at which additional data collection was considered unlikely to lead to the identification of new themes, which may be considered to align with data or thematic saturation (49, 52).

Lines 831-839: As highlighted in the methods, the sample size was guided by the aim of achieving saturation. Saturation is considered an important concept for improving the quality of qualitative research by some researchers, yet it remains inconsistently defined and controversial (47-49). Braun and Clarke (47) have questioned whether saturation is consistent with the principles of reflexive thematic analysis but acknowledge the concept of saturation may be appropriate in some circumstances, such as studies involving a relatively low level of interpretation. In this study, it is possible that using a different approach to determine the sample size, such as information power (76), may have led to further data collection and valuable additional insights.

We achieved the focus group guidelines you suggest, as we held three focus groups with four or five participants each.

24. Why have the authors mixed the participants instead of separating them into pre TKR and post TKR? The authors need to justify why they combined. If this is a limitation – it should be mentioned.

We have now added further justification for this decision.

> Lines 258-263: Participants were primarily allocated to the focus groups based on when they were recruited and their availability/timing preferences, rather than their experience of TKR. This approach was chosen because ensuring the timing of focus groups is convenient for patients may encourage them to participate (54). Furthermore, not all participants had been recruited at the time of the first and second focus groups as the recruitment overlapped with the data collection.

We have also now highlighted this in the limitations section.

> Lines 799-805: Another limitation of the sample was that only one participant was awaiting her first TKR. A further four participants were awaiting TKR surgery on their second knee, but their perspectives are likely to have been influenced by their previous TKR. Allocating participants to focus groups based on their experience of TKR may have provided more insights into whether the perspectives of patients who were awaiting TKR and those who had undergone TKR differed but was not undertaken due to the pragmatic considerations highlighted in the methods.

Data collection

25. Page 9 lines 231-232 – Two PAG PPI members were invited to review the Topic Guide but did not suggest any changes.' Can the authors provide further details. Was a meeting held with them? It is unclear how this review was conducted and if there were any limitations to the process.

We have now provided further details about this.

> Lines 289-292: Two PAG PPI members were invited to review the Topic Guide but did not suggest any changes. The PPI members provided feedback via email as organizing an in-person meeting to discuss the Topic Guide was not feasible within the study timeline.

26. Page 10 Lines 245-245 'The lead researcher recorded field notes during and/or shortly after each focus group.' Can the authors elaborate why they did this and did this assist with their analysis?

We have now elaborated on this.

> Lines 305-310: In line with good practice for qualitative research (43), the lead researcher recorded field notes during and/or shortly after each focus group. Key points recorded in the field notes related to the focus group practicalities (e.g., technical disruptions), group dynamics (e.g., whether all the participants contributed to similar degrees), and overall discussions (e.g., key points emphasized by participants).
>
> Lines 328-330: Field notes were used to assist the analysis where appropriate, with the summary of the overall focus group discussions being particularly useful for ensuring key points emphasized by participants were considered.

27. Focus groups are well known for their drop-out rates – how did the authors prepare for this?

We have now added details of this.

> Lines 250-251: To help build rapport and minimize the risk of dropouts, the researcher discussed the study via telephone with all participants prior to their focus group.

Data analysis

28. Page 11 lines 279-282 – This paragraph confused me. Are the findings that are reported in this study just focused on the focus groups or does the findings include Phase 1 of the VKS? Again, this jump back to the wider project is very confusing.

We have removed this sentence for clarity.

Trustworthiness and reflexivity

Table 1

29. The mention of focus groups under credibility is also missing that focus groups can enhance discussion through the group interaction of participants sharing similar or different views.

We have now added this to table 1.

> Table 1: Focus groups were used to encourage participants to freely express their perspectives and enhance discussions through the group interaction of participants sharing similar or different views

30. How was rapport established?

We have now added details of this in the main text and Table 1.

> Lines 250-251: To help build rapport and minimize the risk of dropouts, the researcher discussed the study via telephone with all participants prior to their focus group.

> Table 1: The researcher discussed the study via telephone with all the participants prior to their focus group to help establish rapport

31. The table again mentions the findings are incorporated with subsequent phases of VKS – Again is this more for the discussion and next steps or did this occur in this study?

We have now clarified this and included a reference for the VKS development paper.

> Table 1: The findings informed the VKS prototype and feedback on the VKS prototype was obtained through think-aloud interviews in a subsequent phase of the VKS project (39)

32. As outlined in point 26 – how did the reflexive journal help?

We have addressed point 26, which refers to field notes, above. We have also expanded how the reflexive journal helped.

> Lines 366-373: The lead researcher was aware that she had developed various preconceptions about potential barriers and facilitators to engagement with the VKS through her clinical work, previous research, PPI activities and wider reading. To minimize the impact of these preconceptions on her interpretation of the data, she employed various strategies to enhance confirmability (Table 1). For example, she used a reflexive journal to record reflections on aspects such as her preconceptions, dual position as a researcher and health professional, key

challenges encountered during the analysis process, and discussions with other research team members.

33. Maximum variation purposive sampling was used to select a diverse range of participants – the result though was not at times diverse in some areas e.g. only White British participants – noted this is mentioned in the limitations but what went wrong with this approach? Further detail is required and what would the authors recommend in future.

As detailed in our methods section, we selected four key purposive selection criteria for this study.

> Lines 223-227: Correspondingly, maximum variation purposive sampling was employed to select participants who varied in key characteristics (45). These comprised age, gender, confidence in using the Internet (unconfident, neither confident nor unconfident, confident, very confident (46)) and experience of TKR (listed for TKR versus undergone TKR).

We achieved our aim of obtaining diversity in these characteristics despite the substantial challenges of recruiting participants during the early stages of the COVID-19 pandemic. We appreciate that the diversity of our sample was limited in certain characteristics and have expanded our discussion of that and how it could be addressed.

> Lines 785-813: This study had a number of limitations, particularly regarding the sample diversity. Participants varied in characteristics such as age, gender and educational level, but only one participant was unconfident in using the Internet and none had a disability/health condition that affected their ability to use a website. Barriers and facilitators related to low digital literacy and accessibility may therefore have been overlooked. Furthermore, all participants were White British. Rates of TKR vary between ethnic groups (70) and qualitative research has identified disparities in Black and White patients' perspectives of post-TKR rehabilitation (71). Correspondingly, patients from different ethnic groups may face different barriers and facilitators to engagement with pre-operative TKR education and prehabilitation. In addition, only patients able to communicate in English were eligible, despite around 2% of the population in England and Wales not being able to speak English well or at all (72). This an important limitation as

patients unable to communicate in English are likely to face additional barriers to accessing TKR care.

Another limitation of the sample was that only one participant was awaiting her first TKR. A further four participants were awaiting TKR surgery on their second knee, but their perspectives are likely to have been influenced by their previous TKR. Allocating participants to focus groups based on their experience of TKR may have provided more insights into whether the perspectives of patients who were awaiting TKR and those who had undergone TKR differed but was not undertaken due to the pragmatic considerations highlighted in the methods.

Due to COVID-19-related restrictions, all the recruitment was undertaken using community approaches such as social media. As well as limiting the sample diversity, this may have led to the recruitment of individuals who were particularly interested in pre-operative TKR education and prehabilitation and/or digital health, increasing the risk of self-selection bias (73). To address these issues in future research, it would be valuable to recruit patients from NHS clinics and include additional purposive selection criteria such as ethnicity. These considerations were addressed in the final phase of the VKS project (39).

Page 12

34. Were the participants aware of the researcher's background? If so, what implications could this have?

We have now clarified that the participants were aware of the researcher's background but that did not appear to have a major impact on the data obtained.

Lines 361-366: All the participants were aware that the researcher was a physiotherapist undertaking a PhD to develop a new pre-operative TKR care website. This did not appear to have a major impact on the data obtained as the participants generally appeared open about sharing their experiences of TKR care, both positive and negative. Similarly, participants were willing to express positive and negative perspectives about pre-operative TKR education, prehabilitation and digital interventions.

Results

35. Please outline in the manuscript why 6 participants were not included – note this is mentioned in the patient flow chart, but detail in this section would be helpful. As the PIS is the only abbreviation in the flow chart, I would spell it out in the box rather than just have it on its own in the key.

We have now added the reasons for not including the six participants directly in the text and removed the PIS abbreviation from Additional File 3.

> Lines 379-382: Reasons for exclusion at the screening stage were not meeting the eligibility criteria (n=1), not meeting the purposive selection criteria (n=4) and declining participation (n=1).

36. There is a lack of detail on the focus group composition in the results. – e.g., the mix of awaiting and having TKR etc.

We have now added details about the compositions of the focus groups.

Lines 387-394: The compositions of the focus groups were as follows:

- Focus group 1: three participants who were awaiting and had undergone TKR, and two participants who had undergone TKR.
- Focus group 2: one participant who was awaiting and had undergone TKR, three participants who had undergone one TKR, and one participant who had undergone TKR on both knees.
- Focus group 3: one participant who was awaiting TKR and three participants who had undergone TKR.

Thematic analysis overview

37. Page 13 lines 318-320 – The findings suggested…digital interventions' is more for the discussion.

We have now removed this sentence from the results.

38. I found the results very hard to follow as the themes and sub themes are repetitive and cross over. The constant reference of barriers/facilitators is also repetitive.

As highlighted in response to point 6, in their book, '[Thematic analysis: a practical guide']' Braun and Clarke (2021) emphasise that themes need to have a central organising

concept, but linkages and relationships between themes and subthemes are appropriate. Both the themes have a central organising concept (Theme 1: accounting for individual differences; Theme 2: tailoring to the pre-operative concept). In addition, we have explained how they intersect in the thematic map and text.

> Lines 566-569: Whilst this theme [Theme 2] encompasses barriers and facilitators that appear to be common in the pre-operative TKR context, the barriers and facilitators do not apply to all individuals and may be experienced to differing degrees. Correspondingly, this theme intersects with Theme 1, *'Accounting for individual differences'*.

The results section repeatedly references barriers and facilitators because the aim of our study was to explore patients' perspectives of potential barriers and facilitators to engagement with the VKS, so the study was focused on identifying barriers and facilitators.

39. Theme 1 - Is the repetition of engagement with each subtheme necessary? Is theme 1 more about engagement that covers exploring the individual differences? Similarly, the digital aspect can be covered under the pre-operative education as a mode of education. It also comes up again under that section.

We believe repeating *'engagement'* is important to show that the subthemes relate to the behaviors. We have now reworded the theme overview to highlight what the theme and subthemes focus on.

> Lines 410-418: Multiple participants from across all three focus groups emphasized that *"everybody's different"*. Differences in participants' individual circumstances and preferences meant their perspectives of potential barriers and facilitators to engagement with the VKS varied. This theme includes three subthemes that demonstrate the impact of individual differences on barriers and facilitators to engagement with digital technologies, pre-operative education and prehabilitation. The subthemes highlight how digital features could address the individual differences where applicable. Overall, this theme suggests that accounting for individual differences would help to optimize patient engagement with pre-operative TKR education and prehabilitation digital interventions.

Whilst digital delivery is relevant to pre-operative education, there are many factors that may influence patient engagement with pre-operative education that are not dependent on the delivery mode. We have now added a quote to demonstrate that point in relation to information about what happens during TKR surgery.

> Lines 484-486: *"I did not want to know at all what they were going to do to me. And anybody who started to tell me, I just switched off, I did not want to know or to see anything, or to read about it or see pictures."* (Dorothy, 70–79, confident, post-TKR)

We have also now highlighted that we did consider combining the barriers and facilitators tables for engagement with pre-operative TKR care in a web-based format and engagement with pre-operative TKR education but decided to keep them separate.

> Lines 341-349: The lead researcher discussed the analysis with research team members at her monthly supervision meetings and via separate meetings/email correspondence as required. These discussions ensured differing perspectives were considered and helped resolve uncertainties that arose during the analysis process. For example, the lead researcher was initially unsure whether to create separate barriers and facilitators tables for engagement with pre-operative TKR care in a web-based format and engagement with pre-operative TKR education or combine them in a single table. Based on team discussions, it was decided to keep them separate because they are distinct concepts and combining them may have resulted in important details being lost.

40. The prehabilitation section was light and the subthemes could benefit from including a brief description. For example, what is meant by prehabilitation?

We have now provided a more detailed description of prehabilitation to the introduction.

> Lines 94-97: Prehabilitation involves optimizing patients' health and wellbeing prior to surgery with the aim of improving their post-operative recovery (14, 15). A multimodal approach to prehabilitation is generally advocated, which may include various strategies such as exercise programs, weight management, and alcohol reduction (14, 15).

We have also expanded the prehabilitation section in the results.

Lines 509-554: As for engagement with digital technologies and pre-operative education, participants' individual circumstances and preferences affected their perspectives of barriers/facilitators to engagement with prehabilitation. Differences in participants' preferred exercise types and delivery modes appeared to be particularly important. For example, one participant reported finding exercise classes motivating, whereas another indicated that she does not like the constraints of exercise classes:

> *"Because I'm not good at following patterns, I'm not a, you know, I don't like to have to, I don't like classes, you know, I don't like the routine, but I do like the exercises. So doing them as I felt like it, would work for me better. Just different people have different ways of doing it."* (Rosie, 60–69, very confident, pre- and post-TKR)

Lifestyle choices (e.g. being vegan), personal characteristics (e.g. a determined personality), and other health issues (e.g. heart problems) were all perceived to affect engagement with prehabilitation:

> *"So I was all prepped up ready and then they said hang on, your heartrate's too low. That's because of all the physical exercise I do. So I had to slow down my exercise so they were going to go back four months later and have my operation done then."* (Cameron, 60–69, very confident, post-TKR)

Participants in one group suggested signposting patients to healthy eating advice on existing credible websites, as they felt that would account for individuals' differing needs whilst avoiding unnecessary duplication of online information:

> *"And some people would say, you know, I can't do this, or I've got that, and if you just referred them to the NHS page, it covers pretty much, basically everything, and all different, you know, eventualities of being vegan, or you know, diabetic, or anything like that."* (Sophia, 40–49, very confident, post-TKR)

Additional barriers to engagement with prehablitation related to participants' differing environmental circumstances. Lack of access to specific equipment/facilities was identified as a barrier to engagement with exercise:

> *"I'd been going to the gym four or five times a week, and swimming, but then obviously lockdown happened, and I've not done as much."* (Molly, 40–49, very confident, pre-TKR)

Correspondingly, participants in one focus group emphasized the value of exercises that require household items only. Another environmental circumstance that presented a barrier to engagement with prehabilitation was going on holiday pre-operatively:

> *"The one thing that I did do differently, and it was particularly difficult for me, because we went on holiday, got back about ten days before the operation, and it was cutting down on alcohol. Now, I'm not an alcoholic, but I do like my wine at weekends, and on holiday."* (Irene, 50–59, confident, post-TKR)

41. Theme 2 was just further evidence for what had already been discussed in theme 1.

Theme 2 is distinct from Theme 1 as it focuses on barriers and facilitators that specifically relate to pre-operative contextual features. We have amended the theme description to help demonstrate that and how it intersects with Theme 1.

> Lines 557-569: Many of the reported barriers and facilitators to engagement with the behaviors targeted by the VKS appeared to relate closely to the pre-operative context. Key pre-operative contextual features included physiological/psychological factors, social/occupational factors, and limitations in pre-operative TKR care provision. This theme includes three subthemes that highlight barriers/facilitators and associated digital features related to these pre-operative contextual features. Overall, this theme suggests that tailoring pre-operative TKR digital interventions to these contextual features would help to optimize patients' engagement with them.

> Whilst this theme encompasses barriers and facilitators that appear to be common in the pre-operative TKR context, the barriers and facilitators do not apply to all individuals and may be experienced to differing degrees. Correspondingly, this theme intersects with Theme 1*, 'Accounting for individual differences'.*

42. I would recommend the authors to review the themes and rewrite this result section. The narrative is not clear.

We have rewritten aspects of the results section as explained in our responses to your other points and added numerous additional quotations as recommended by reviewer 1. We do not feel further re-writing is appropriate given the positive feedback we have received from reviewers 1 and 3.

43. It is just briefly mentioned that the analysis was discussed with other research team members – what was the frequency of these meetings and how did this help with the analysis? Can be linked in with the reflexive diary and notes etc.

We have added details of the meetings to the methods section.

> Lines 341-349: The lead researcher discussed the analysis with research team members at her monthly supervision meetings and via separate meetings/email correspondence as required. These discussions ensured differing perspectives were considered and helped resolve uncertainties that arose during the analysis process. For example, the lead researcher was initially unsure whether to create separate barriers and facilitators tables for engagement with pre-operative TKR care in a web-based format and engagement with pre-operative TKR education or combine them in a single table. Based on team discussions, it was decided to keep them separate because they are distinct concepts and combining them may have resulted in important details being lost.

We have also linked this to the description of the reflexive journal.

> Lines 370-373: For example, she used a reflexive journal to record reflections on aspects such as her preconceptions, dual position as a researcher and health professional, key challenges encountered during the analysis process, and discussions with other research team members.

44. Showing a thematic map of the analysis process would help to understand how the themes have been decided upon and the iterative process. The current thematic map is just the overall findings.

We have presented the overall findings as a thematic map to provide a clear summary of the findings and how the themes and subthemes relate to each other, which is a commonly used approach in qualitative studies employing reflexive thematic analysis. As you have highlighted, reflexive thematic analysis is an iterative process. We do not feel it would be appropriate or feasible to attempt to capture the entire analysis process in a diagram, and trying to capture some elements of the analysis in a diagram is likely to cause confusion. In addition, we have reviewed numerous qualitative papers recently published in *BMC Musculoskeletal Disorders* and have not found any in which that approach has been used. We appreciate your point about understanding how the themes have been decided on and have added details of a key change to the methods as an example of how we collapsed themes.

> Lines 322-326: Codes were clustered into candidate themes/subthemes, which were critically reviewed. This led to redevelopment of all the themes/subthemes and collapsing of some themes/subthemes. A key change was collapsing two candidate themes, *'Tailoring to pre-operative phase barriers' and 'Targeting key facilitators'*, into a single theme, *'Tailoring to the pre-operative phase'*.

Barriers and facilitators table

45. There is a disconnect with the table and the results for me. I'm not sure what the flow is here. Is this table a summary all the barriers/facilitators to take forward applied for the VKS? I note the analysis last step covers the table but its not mentioned how this integrates with the findings.

We have now clarified this in the analysis section and signposted to the paper reported how the findings were used to inform the VKS.

> Lines 334-339-: In addition to the narrative report, tables were created to summarize the barriers, facilitators and digital features from across both themes. Separate tables were created for each behaviour targeted by the VKS. This approach was based on a previous evidence-, theory- and person-based intervention planning study (60) and ensured that the data were optimally

structured for informing the subsequent project phases, which are described in Anderson et al. (39).

Discussion

46. The discussion reverts to barriers and facilitators which adds to the confusion of point 46. Perhaps the findings should have a summary of the main barriers and facilitators linked with the analysis and that the barriers and facilitators table shows how this has been adapted for the VKS going forward?

We have discussed barriers and facilitators in the discussion because the aim of this study was to explore patients' perspectives of potential barriers and facilitators to engagement with the VKS. The main barriers and facilitators we identified are summarised in the findings, and the full lists of barriers and facilitators are in Tables 3 to 6. We have now included a reference to the paper reporting how this study's findings were used in the development of the VKS.

> Lines 865-866: This study's findings have informed the development of a VKS prototype as detailed in Anderson et al. (39).

47. The authors cover the main aspects of the discussion, but I would need to review again once the actual study is clearer for me.

We hope our amendments have now clarified the overall study.

Tables

48. Table 2 – The experience of TKR it is listed as pre, post, pre, post and post x2 – unsure of these categories – did some participants have more than one knee operated on?

We have now clarified that in the table footnotes.

> Table 2 footnotes: *Post x 2*, previously undergone TKR on both knees

Reviewer 3 comments and responses

This is a well written piece, it is evident significant time was placed on the writing and editing and proof reading of the submission, thank you for this.

It a nice small scale study, as part of a PhD, which presents some interesting findings, it is important to not overstate the findings of this qualitative study, given the n=14, and the highlighted limitations in the recruitment, the sample demographic and the diversity in pre/post op particulars.

Thank you very much for your positive and helpful feedback. We appreciate your point about the importance of not overstating the findings. We have updated the wording of the discussion section by removing the word *'important'* from before *'implications'* using *'may'* as you have suggested below.

> Lines 843-849: The limitations in pre-operative TKR care provision identified in this study may have implications for clinical practice. The findings suggest improvements may be needed in a range of areas, including provision of pre-operative TKR exercise programs and education on topics such as how to practically prepare for surgery. This study also identified issues related to the timing of pre-operative TKR care delivery, corresponding with a previous UK-based study (77). Digital interventions may be a promising approach for addressing these issues as they could provide rapid access to pre-operative TKR education and prehabilitation support.

Some revisions i suggest
Line 79: you say "are older adults living with overweight or obesity", I would suggest rewording "living with overweight".

We have now updated this text based on your feedback and feedback from reviewer 1.

> Lines 82-86: Key risk factors for OA include increasing age, previous knee injury, female gender, and being overweight or obese (5). Correspondingly, the average age of patients undergoing TKR is 70 years old, approximately 56% of patients undergoing TKR are female, and approximately 90% are overweight or obese (6, 7).

Line 80: are you referring to public patients with regards to waiting times, this should be made clear if so.

We have now clarified which patients we are referring to.

> Lines 86-87: In the English National Health Service (NHS), patients often face a long wait for TKR surgery (8).

Line 244, where you report time of focus groups, I suggest this is a result and not a method and thus should be moved to the results.

We have now moved the following sentence to the results section.

> Line 396: The focus groups lasted between 95 and 110 minutes.

Line 305: should this read Figure and not File?

We have now clarified this sentence.

> Lines 381-382: The flow chart in Additional File 3 provides further details of the flow of patients in the study.

Line 629: in light of the limitations mentioned in the section prior to this, using words like may/could would be better suited to this implications paragraph, so as not to overstate the findings of the study or its subsequent implications.

We have removed the word *‘important’* from before *‘implications’* and added *‘may’* as you have suggested.

> Lines 843-849: The limitations in pre-operative TKR care provision identified in this study may have implications for clinical practice. The findings suggest improvements may be needed in a range of areas, including provision of pre-operative TKR exercise programs and education on topics such as how to practically prepare for surgery. This study also identified issues related to the timing of pre-operative TKR care delivery, corresponding with a previous UK-based study (77). Digital interventions may be a promising approach for addressing these issues as they could provide rapid access to pre-operative TKR education and prehabilitation support.
