# Supplementary material for: “I’m a paper and pencil person”: a qualitative descriptive study of potential barriers and facilitators to engagement with pre-operative total knee replacement education and prehabilitation digital interventions
Source: BMC Musculoskelet Disord. 2025 Jul 4;26:652. doi: 10.1186/s12891-025-08673-1 (PMC12228215; doi:10.1186/s12891-025-08673-1)
Supplement: Supplementary file 5 — Supplementary Material 5: Reviewers’ reports [file 12891_2025_8673_MOESM5_ESM.zip › Second_Response_To_Reviewers_VKS2_BMC_MSKD_1.0_09Jul24.pdf]

Point-by-point response to reviewers – second revision

Manuscript details

Title: "“I’m a paper and pencil person”: a qualitative descriptive study of potential barriers and facilitators to engagement with pre-operative total knee replacement education and prehabilitation digital interventions"
Submission ID: af87cc7c-f6d8-4382-996a-a8f9367995ba
Journal: BMC Musculoskeletal Disorders

Editor comments and response

Thank you for revising your manuscript in accordance with the reviewers’ comments. After careful evaluation, we would like to request further revisions to your manuscript. Please find below the reviewers' comments for you to review and address accordingly.

Many thanks for the opportunity to revise our manuscript further. We have reviewed all the reviewers’ comments and ensured that we have taken a balanced approach for addressing the contrasting reviews. The line numbers provided below refer to the tracked changes version of the manuscript with the track changes shown.

Reviewer 1 comments and response

Thank you for the opportunity to review your revised manuscript.
I am satisfied that your revision addresses the concerns i raised in my original review (and those of the second reviewer)

Thank you for this positive feedback on our manuscript. We are pleased you are satisfied that we have addressed your concerns and those of Reviewer 2.

Reviewer 4 comments and response

"PEER REVIEWER ASSESSMENTS:

OBJECTIVE - Full research articles: is there a clear objective that addresses one or several testable research questions? (Brief or other article types: is there a clear objective?)
Yes - there is a clear objective

DESIGN - Is the current approach (including controls and analysis protocols) appropriate for the objective?
Yes - the approach is appropriate

EXECUTION - Are the experiments and analyses performed with sufficient technical rigor to allow confidence in the results?
Yes - experiments and analyses were performed appropriately

STATISTICS - Is the use of statistics in the manuscript appropriate?
Yes - appropriate statistical analyses have been used in the study

INTERPRETATION - Is the current interpretation/discussion of the results reasonable and not overstated?
Yes - the author's interpretation is reasonable

OVERALL MANUSCRIPT POTENTIAL - Has the author addressed your concerns sufficiently for you to now recommend the work as a technically sound contribution? If not, can further revisions be made to make the work technically sound?
Yes - current version is technically sound

PEER REVIEWER COMMENTS:

GENERAL COMMENTS: The reviewers of the previous assessment highlighted a number of important points. Overall, this has resulted in a comprehensive re-writing of the paper. The result is a robust study reported clearly and justified throughout.

The authors have comprehensively addressed the points raised by the two reviewers. The response to Reviewer 1’s comments on MRC framework positioning, saturation, and addition of the paragraph on digital health literacy were particularly clear. The justification for not using the term ‘emerged’ (as suggested by Reviewer 1) and using the term ‘intersecting’ (as suggested by Reviewer 2) is justified from a methodological perspective and is reasonable. This is also clearly explained in the revised paper. Reviewer 2’s comment on sampling people awaiting as well as those who had undergone knee replacement was also addressed in the paper and explored further within the limitations section. The authors have offered more information on the context of the study, both setting and population, to address Reviewer 2’s concern appropriately. A clearer description of the Project Advisory Group and PPI input has also been offered

and weaved within the methods (thereby addressing an important point raised by Reviewer 2). Finally, Reviewer 3's minor points have been comprehensively addressed within the revision.

I am confident that that authors have addressed all points appropriately, the changes are reflective in the paper, and I have no further comments to raise. The addition of the supplementary files and supporting images/figures provide important context which is incredibly helpful. The paper has been robustly reported, following methodological approaches of Braun and Clarke. The Discussion remains pertinent and appropriately interprets the results from this study, within the context of previous understanding and literature. I have nothing to add after my additional critique.

In summary, I believe this to be a robust piece of work which offers important insights into the topic and fully support its acceptance for publication.
ADDITIONAL REQUESTS/SUGGESTIONS:
Nothing additional"

Thank you for this positive feedback on our manuscript and recommending it for acceptance. We really appreciate you undertaking a thorough review of our manuscript and providing detailed feedback on our revisions in relation to the points raised by the other reviewers.

Reviewer 2 comments and responses

Manuscript title: "I'm a paper and pencil person": a qualitative descriptive study of potential barriers and facilitators to engagement with pre-operative total knee replacement education and prehabilitation digital interventions.

Submission ID: af87cc7c-f6d8-4382-996a-a8f9367995ba

Thank you to the authors for revising the manuscript and for taking some of the previous recommendations on board. The manuscript is much improved with its transparency. However, there are still a number of queries/points that need to be addressed.

Thank you for taking the time to review our revised manuscript and provide further feedback. We have carefully considered all your queries/points alongside the positive feedback of the other reviewers. We have implemented all the suggested changes that we feel add value to the manuscript.

Abstract

1. I note that my previous recommendation to include Patient and Public Involvement (PPI) in the methods of the abstract has not been done nor addressed why it has not been included in the authors response – (Previous point 10.) The authors response to this point is in reference to my later point of PPI in the methods in the main paper. I recommend the authors add PPI to the abstract method.

We apologise that we misunderstood your original recommendation as referring to the methods section of the main text rather than the abstract. We have now added a sentence about PPI to the abstract and amended other aspects of the abstract to ensure that it remains within the limit of 350 words specified by BMC Musculoskeletal Disorders.

> Lines 39-41: Three Patient and Public Involvement representatives were involved in aspects such as reviewing the participant documents and/or plain English summary.

Methods

Design

2. Thanks to the authors for linking in their publication in regards to my previous comment (Previous Point 15). However, it is important to make it easy for the reader and some may not have the time to explore the other publication. As the paper stands, it is not clear how this study fits and what the other phases are. It should not be expected for the reader to look up the other paper. It is also a missed opportunity for promoting where this study fits in the overall picture and the work that has been done in this area. I have looked at this paper the authors have referenced and note this is a great diagram. I would again recommend the authors provide a more succinct/basic diagram of where the study sits and refer to their publication for further information. The sentence on Lines 160-161 could instead state: The overall VKS development project and where this study fits is shown in Figure X, Phase 2. This is a mixed…. (brief sentence). Further detail is reported in Anderson et al. (39). Alternatively, the authors could include a Table briefly describing the phases of the overall study to provide the background detail and refer to the reference for further detail.

We have now included the diagram from our other publication with the following legend:

> Lines 1213-1217: **Figure 1: Flowchart of the overall Virtual Knee School development project**
> This figure has been reproduced from Figure 1 in Anderson et al. (39) without any changes under the terms of the Creative Commons Attribution License (https://creativecommons.org/licenses/by/4.0/).
> Alternative text for Figure 1 is available in Additional File 1.

We have included an Additional File with alternative text of Figure 1. We have also amended the text in the main manuscript in line with your suggestions:

> Lines 160-165: The overall VKS project employed a complex mixed methods design, primarily underpinned by pragmatism (38). This study was Phase 2 of the VKS project. Figure 1 summarizes the overall VKS development project and where this study fits. Further details of the VKS project are reported in Anderson et al. (39).

Project Advisory Group and Patient and Public Involvement

3. Please amend the title "Project Advisory Group and Patient and Public Involvement' to just PPI. Project advisory groups are one approach of PPI, as are using PPI representatives (although I am not entirely sure who the PPI representatives are – see later point).

This section explains the role of the Project Advisory Group and PPI representatives. The Project Advisory Group included both professional representatives and PPI representatives, as explained in the existing text:

> Lines 193-198: The PAG included an independent chair, a collaborator from a local association of acute NHS trusts, three Patient and Public Involvement (PPI) representatives with diverse lived experiences relevant to the study (one of whom was recruited after this study), the lead researcher (AMA) and three of her supervisors.

Amending the title to PPI only would therefore not be appropriate.

4. Lines 199-200 'Two PPI representatives….which included the findings of this study' please add 'preliminary' before findings.

The presentation was given after the completion of this study and included a summary of the study findings. We have not made any substantial changes to how the findings are structured or presented since the presentation, so we do not feel adding ‘preliminary’ here is appropriate.

Participants

5. Lines 203-204 – Project Advisory Group can be amended to PAG as this has already been abbreviated.

We have updated this text as recommended.

> Lines 211-212: The recruitment strategy and eligibility criteria were discussed and finalized with the PAG.

6. Line 206-207 ‘Phase 1 of the VKS project’ and line 217 – ‘Phases 1 and 4.’ These statements highlight the need to have some background information on the overall study. Please refer to point 2.

As explained above, we have now included a figure summarising the overall VKS development project (Figure 1) and associated additional file (Additional File 1).

7. Whilst I agree on confidentiality, it is appropriate to advise if your PPI representatives have lived experience of the condition you are exploring. Is this not the case? If your PPI representatives do have lived experience of knee replacement/knee osteoarthritis, I would mention this. It is important to be transparent and include the background of the representatives.

We have now added further details about the PPI representatives:

> Lines 197-198: The PPI representatives brought a range of relevant lived experiences to the study, such as lived experience of TKR and barriers to using digital technologies.

8. Line 289 – ‘Two PAG PPI members’ the authors need to be consistent with the terms they use, at other times in the manuscript these members are referred to as PPI representatives. It is important to be consistent with terminology.

We have updated the text to ‘PPI representatives’ as suggested.

> Lines 305-310: Two PPI representatives were invited to review the Topic Guide. The PPI representatives responded via email as organizing an in-person meeting to discuss the Topic Guide was not feasible within the study timeline. Neither of the PPI representatives suggested any changes to the Topic Guide, so no changes were made.
>
> Lines 316-318: These consisted of 11 examples of website features developed based on the findings of Phase 1 of the VKS project (34, 44) and PPI representative feedback.

Data collection

9. Line 257 ‘…eConsent and Questionnaire..’ what did the questionnaire involve or is this just collecting demographic information? Further detail is required.

We have added a sentence to clarify this.

> Lines 268-270: The questionnaire was used to collect data about the participants’ characteristics, such as their ethnicity.

10. Lines 266-268 ‘No co-facilitators were involved due to the small size of the focus groups and to ensure that participants could be offered maximum flexibility with the focus group scheduling.’ Depending on the guidelines used typical focus group can consist of e.g. 6-8 participants. Adding how you define the number of participants would help to be clear on why the authors are stating these are small groups.

We have now added further details about the number of participants here.

> Lines 278-280: No co-facilitators were involved due to the small size of the focus groups (four to five participants per group) and to ensure that participants could be offered maximum flexibility with the focus group scheduling.

11. The authors response to my previous review (Previous Point 25), Lines 289-292: ‘Two PAG PPI members were invited to review…timeline’ I would recommend rewording this sentence as it seems to contradict. The first sentence states no feedback, and the second states feedback was via email. This could be amended to ‘Two PAG PPI

members were invited via email to review the Topic Guide and no changes were made from their reviews.’ Again, some background on who these individuals were and their expertise is needed.

We have now amended this text for clarity:

> Lines 305-310: Two PPI representatives were invited to review the Topic Guide. The PPI representatives responded via email as organizing an in-person meeting to discuss the Topic Guide was not feasible within the study timeline. Neither of the PPI representatives suggested any changes to the Topic Guide, so no changes were made.

12. With regards to my previous comment (Previous Point 27) on focus groups and drop-out rates, the authors have stated that rapport was built on telephone contact discussing the study that helped minimise the risk of dropout. This is standard practice for any study to discuss the project with potential participants. Was there anything in particular during these discussions that helped with the rapport building that minimised drop out risk? If so, this would be interesting for other researchers to learn from.

We agree that discussing the study with participants is standard practice, as well as being a valuable step for helping to establish rapport. We do not have any additional points to report related to this, but we have updated our text to highlight our approach was in line with standard practice.

> Table 2: In line with standard practice, the researcher discussed the study via telephone with all the participants prior to their focus group to help establish rapport

13. Lines 300-301 ‘The trigger materials were emailed to the participants prior to their focus group and shared on screen during each focus group.’ How was the screen sharing dealt with the participants on the telephone? Were the participants on the telephone directed to review the material emailed to them at this point?

We have now amended the text to confirm this.

> Lines 318-322: The trigger materials were emailed to the participants as a Portable Document Format (PDF) document prior to their focus group and shared

on screen during each focus group. Participants who joined the focus groups via telephone were directed to review the PDF document provided via email when the trigger materials were shared on screen.

Data analysis

14. Regarding my previous review (Previous Point 23), whilst the authors state 'We are aware that Braun and Clark (2021) have questioned the use of saturation in reflexive thematic analysis, although they state 'Our point here is not that data saturation is never valid and never a useful concept' please note that Braun and Clark refer to this sentence in regards to 'some forms of thematic analysis' and 'is best avoided' in reflexive thematic analysis. I am unsure what the authors mean by low level interpretation and how this fits with reflexive thematic analysis?

As explained in our manuscript, we appreciate saturation is a contentious issue and Braun and Clark have questioned its appropriateness in the context of reflexive thematic analysis. We assume your reference to 'is best avoided', is referring to the following sentence, which does not present definitive guidance:

> 'And for reflexive TA, data saturation is an awkward if at times convenient bedfellow, though one perhaps best avoided.'

As we have highlighted in our manuscript, there is no universally accepted alternative for determining sample size in qualitative studies. Correspondingly, saturation remains a widely used concept within qualitative research.

We have provided examples below of studies published in BMC Musculoskeletal Disorders, which have used the concept of saturation to help determine the sample size and analysed the data using reflexive thematic analysis.

Moore, A., Wylde, V., Bruce, J. et al. Experiences of recovery and a new care pathway for people with pain after total knee replacement: qualitative research embedded in the STAR trial. BMC Musculoskelet Disord 23, 451 (2022). https://doi.org/10.1186/s12891-022-05423-5

Chua, H., Brady, B., Farrugia, M. et al. Implementing early mobilisation after knee or hip arthroplasty to reduce length of stay: a quality improvement study with embedded

qualitative component. BMC Musculoskelet Disord 21, 765 (2020). https://doi.org/10.1186/s12891-020-03780-7

Tutuhatunewa, E.D., Stevens, M., Dams, O.C. et al. Exploring patient satisfaction after operative and nonoperative treatment for midshaft clavicle fractures: a focus group analysis. BMC Musculoskelet Disord 21, 560 (2020). https://doi.org/10.1186/s12891-020-03557-y

Watkins, R., Young, G., Western, M. et al. Nobody says to you “come back in six months and we’ll see how you’re doing”: a qualitative interview study exploring young adults’ experiences of sport-related knee injury. BMC Musculoskelet Disord 21, 419 (2020). https://doi.org/10.1186/s12891-020-03428-6

None of the above published studies published in BMC Musculoskeletal Disorders acknowledge the controversy or limitations of the concept of saturation. In contrast, we acknowledged the controversy and limitations of the concept of saturation in our original submission, and substantially expanded our discussion of saturation in our revised manuscript. We have now also added an explanation about what ‘relatively low level of interpretation’ means.

> Lines 251-253: In line with this study’s qualitative descriptive design, it involved a relatively low level of interpretation (i.e., the focus was on ensuring that the findings closely reflected the participants’ own perspectives).

Given this is not a methodological paper and we have already gone well beyond the usual level of detail included about saturation in papers published in BMC Musculoskeletal Disorders, we do not feel that going into any further detail about this is appropriate or justified.

Results

Participants

1. Lines 377-378 – 28 patients contacted the lead researcher and 23 were screened – what happened to the other 5? Was it a case of lost contact?

This is reported in the flowchart in Additional File 4 (previously Additional File 3):

> **Not screened (n=5)**

Not comfortable participating in a telephone/online discussion (n=1)
No response following provision of the PIS (n=4)

2. I presume pseudonyms have been used for the participants? Please add a sentence in this section outlining this. It would also be worth explaining the confidence level (provided by the participant? Was this part of the questionnaire?) and age are also listed with the names.

We had previously explained that we had used pseudonyms:

> Lines 418-420: The illustrative quotes below are labelled with the participant's pseudonym, age group, confidence in using the Internet and experience of TKR surgery.

As explained in our methods, confidence in using the Internet was one of the purposive selection criteria. We have now confirmed that that information was self-reported and collected at the screening stage:

> Lines 231-235: Correspondingly, maximum variation purposive sampling was employed to select participants who varied in key characteristics (45). These comprised age, gender, self-reported confidence in using the Internet (unconfident, neither confident nor unconfident, confident, very confident (46)) and experience of TKR (listed for TKR versus undergone TKR). Participants were asked about these characteristics by the lead researcher at the screening stage.

Thematic analysis overview

3. I am still struggling with the themes. The authors have the detail but the current format lacks clarity. Theme 1's subthemes - as mentioned in my last review, the repetition of 'Engagement' in each of the subtheme title is not needed and could imply whether a thorough analysis has been undertaken. I would suggest the authors amend Theme 1's title to 'Accounting for individual differences with engagement needs' and remove engagement for each of the subtheme titles. With the amended title and the description of the theme, engagement is outlined.

We appreciate you sharing your views about the themes/subthemes. We have carefully considered your suggestions here and in point 4 below. However, given the positive

feedback from the other reviewers, we do not feel it is appropriate to change the themes/subthemes at this stage.

4. Theme 2 ‘Tailoring the pre-operative context’ could benefit from being revised. 2a and 2b subtheme titles could be improved. There is some interesting detail in this theme overall and with some revision it has the opportunity to be clearer. Reviewing the three subthemes there is overlap. The authors highlight in 2a providing alternative strategies, tackling misconceptions, understanding the importance of prehabilitation. 2b includes exercise reminders, feedback from others (healthcare professionals and peers). 2c includes importance of prehabilitation, strategies and timing of the preoperative information. These areas can be further condensed, e.g. timing and tackling misconceptions could go into understanding the importance of prehabilitation information. Exercise reminders could come under alternative strategies. For example, the subthemes could potentially be ‘understanding the importance of the prehabilitation, providing alternative strategies, feedback from others to avoid repetition across subthemes.

As explained above, we do not feel changing the themes/subthemes is appropriate given the positive feedback from the other reviewers.

5. Thematic maps are often used to show the relation between codes to themes and the process of the interpretation of the research findings. Thematic maps can be included for this reason. Please rename ‘thematic map’ as ‘Key themes and subthemes’ as it does not outline the process or an example of how you came to the final themes.

We have now renamed the figure.

> Lines 1235-1237: **Figure 3: Themes and subthemes**
> TKR, total knee replacement
> Summary of the two main themes and six subthemes.

6. Line 424 ‘a mobile app’ please add ‘application’ as this hasn’t been abbreviated. Same with line 120 ‘smartphone apps.’

We have now updated these sentences.

> Lines 444-446: Most participants liked the idea of a pre-operative TKR website, with participants in one focus group suggesting that a mobile application version would be valuable.
>
> Lines 112-113: For example, the quality of TKR smartphone applications and YouTube videos is highly variable (22, 23) and the readability of online TKR education materials is often above the recommended level (24).

7. Lines 425-427 ‘and highlighted …TKR’ could me moved to after the paper and pencil person quote to sum these quotes. E.g. Considering the older demographic of patients undergoing TKR, these extracts highlight that the limited experience of using digital tools is an important barrier for some of the older patients.

This change would mean the quotes about limited experience of using digital tools would follow a sentence about a mobile application being valuable, which we feel would be confusing and out of line with the approach we have used in the rest of the results section, so we have not made this suggested change.

8. Line 436 ‘Dorothy and other participants’ unsure why you single out Dorothy as there is no quote. Perhaps amend to some of or the majority of patients….

We have singled out Dorothy because this sentence directly follows a quote from Dorothy highlighting her reluctance to use digital technologies. We feel it is helpful context for readers to know that Dorothy was one of the participants who was more willing to use a website if it was from a UK-based credible source, with features that are quick and simple to use; therefore, we have not made this change.

9. Line 448 ‘ In contrast, participants in the third focus group…’ Please amend ‘third’ to ‘another’ for confidentiality reasons and not linking the data to a particular group.

We have amended this text as suggested.

> Lines 470-472: In contrast, participants in another focus group preferred animations, with one reporting a negative experience of a real-life video in a digital video disc (DVD) provided by her care team:

10. Lines 451-422 ‘Participants’ individual circumstances and preferences strongly influenced their perspectives of barriers and facilitators to engagement with digital technologies.’ Lines 468-469 ‘Participants’ differing circumstances and preferences affected their willingness to engage with pre-operative education.’ Lines: 509-511 ‘As for engagement with digital technologies and pre-operative education, participants’ individual circumstances and preferences affected their perspectives of barriers/facilitators to engagement with prehabilitation.’ I would recommend removing these sentences as they are repetitive and this is covered in the overall theme description.

These three sentences provide a concise introduction to each subtheme and relate to different behaviours - engagement with digital technologies, engagement with pre-operative education, and engagement with prehabilitation. We therefore feel they are helpful for clarity so have not removed them.

11. Lines 566-569 could be removed if the theme is revised as outlined in point 15.

We are not sure how these sentences relate to point 15 as they explain how the themes intersect. We have decided not to remove these sentences as Reviewer 4 specifically highlighted that our use of the term ‘intersecting’ is appropriate and clearly explained:

> ‘The justification for not using the term ‘emerged’ (as suggested by Reviewer 1) and using the term ‘intersecting’ (as suggested by Reviewer 2) is justified from a methodological perspective and is reasonable. This is also clearly explained in the revised paper.’

Tables

12. Tables 3-6 still are still disconnected for me. Some items are not presented in the findings e.g. ‘family member support?’ How were the VKS design features decided upon - This study, PAG, Research team? Further detail is needed.

The themes and subthemes text summarises all the barriers and facilitators to draw out key considerations. Including every single item in the text would not be feasible without making the paper excessively long. The design features reported in the tables are only those that were discussed in the focus groups as that is what this paper is reporting. Further details of the features included in the VKS and how they were identified are

reported in the overall VKS development paper (Anderson et al. 2023), which the methods section signposts readers to.

13. Table 3 the last facilitator on the first page listed seems more like a barrier? ‘Lack of timely access to adequate pre-operative TKR education and prehabilitation from other sources’.

The reason this has been classed as a facilitator is explained in the results.

> Lines 690-694: Participants’ experiences of pre-operative TKR care were highly varied. Whilst some had positive experiences, many participants highlighted limitations in the pre-operative care they received. These limitations presented barriers to participants’ pre-operative preparations. Being unable to access adequate pre-operative support through other sources also appeared to be a potential facilitator to engagement with a pre-operative TKR website.

14. Table 3 - Why also are some of the barriers/facilitators in the VKS design column not discussed ‘none discussed’. Further detail in this ‘barriers and facilitators tables’ section is required.

We have now justified this in the methods.

> Lines 299-302: The focus groups also explored digital features that could address the above barriers and facilitators. Digital features were not discussed for every individual barrier and facilitator, as that would have required the lead researcher to interrupt the natural flow of the discussions and resulted in the focus groups being excessively long.

15. I suggest revising the header to Summaries of the barriers and facilitators.

The tables provide a brief overview of the barriers and facilitators to we have updated all the titles accordingly.

> Line 1265: Table 3: Overview of barriers and facilitators to engagement with pre-operative total knee replacement care in a web-based format

> Lines 1269: Table 4: Overview of barriers and facilitators to engagement with pre-operative total knee replacement education

> Lines 1272: Table 5: Overview of barriers and facilitators to engagement with a pre-operative total knee replacement exercise program

> Lines 1275: Table 6: Overview of barriers and facilitators to engagement with making pre-operative healthy lifestyle changes

Discussion

16. Lines 468-769 ‘ These findings suggest that online TKR discussion groups could be valuable but need to be appropriately moderated.’ Please add literature to help back this point.

As stated in our discussion, we have made this suggestion based on the findings of our study (rather than other literature). We have now added further explanation of why the findings indicate that moderation may be needed for online TKR discussion groups.

> Lines 787-792: Conversely, concerns about making comparisons with other patients and hearing negative experiences of TKR surgery were identified as barriers to engagement with pre-operative TKR education. These findings suggest that online TKR discussion groups could be valuable but may need to be moderated to help ensure that inappropriately negative views are not shared.
